# A Review on the Application of Deep Eutectic Solvents in Polymer-Based Membrane Preparation for Environmental Separation Technologies

**DOI:** 10.3390/polym16182604

**Published:** 2024-09-14

**Authors:** Gorka Marco-Velasco, Alejandro Gálvez-Subiela, Ramón Jiménez-Robles, Marta Izquierdo, Amparo Cháfer, José David Badia

**Affiliations:** Research Group in Materials Technology and Sustainability (MATS), Department of Chemical Engineering, School of Engineering, University of Valencia, Avinguda de la Universitat, 46100 Burjassot, Spain; gorka.marco@uv.es (G.M.-V.); alejandro.galvez@uv.es (A.G.-S.); ramon.jimenez@uv.es (R.J.-R.); marta.izquierdo-sanchis@uv.es (M.I.)

**Keywords:** deep eutectic solvents (DESs), polymer membrane, pore formation, supported liquid membrane (SLM), environmental separation technologies

## Abstract

The use of deep eutectic solvents (DESs) for the preparation of polymer membranes for environmental separation technologies is comprehensively reviewed. DESs have been divided into five categories based on the hydrogen bond donor (HBD) and acceptor (HBA) that are involved in the production of the DESs, and a wide range of DESs’ physicochemical characteristics, such as density, surface tension, viscosity, and melting temperature, are initially gathered. Furthermore, the most popular techniques for creating membranes have been demonstrated and discussed, with a focus on the non-solvent induced phase separation (NIPS) method. Additionally, a number of studies have been reported in which DESs were employed as pore formers, solvents, additives, or co-solvents, among other applications. The addition of DESs to the manufacturing process increased the presence of finger-like structures and macrovoids in the cross-section and, on numerous occasions, had a substantial impact on the overall porosity and pore size. Performance data were also gathered for membranes made for various separation technologies, such as ultrafiltration (UF) and nanofiltration (NF). Lastly, DESs provide various options for the functionalization of membranes, such as the creation of various liquid membrane types, with special focus on supported liquid membranes (SLMs) for decarbonization technologies, discussed in terms of permeability and selectivity of several gases, including CO_2_, N_2_, and CH_4_.

## 1. Context

The emission of greenhouse gases (GHGs) into the atmosphere has led the world to a severe situation because of global warming and climate change, which also provoked water scarcity for many countries. GHGs are vastly represented by carbon dioxide (CO_2_) emissions, about 79.4%, followed by methane (CH_4_), which represents 11.1% of emissions, but has more than 28 times the greenhouse effect potential of CO_2_ [1]. With respect to CO_2_ emissions, they can be directly derived either from the combustion of fossil fuels, which usually are called anthropogenic emissions, or from several other processes, such as biogas production; in this case, they are mixed with methane [2]. Also, extreme weather conditions might cause water scarcity in the near future [3]. As a consequence, the mitigation of GHGs and the potabilization of water are currently pressing concerns worldwide. Thus, the growing demands for sustainable and efficient environmental technologies have spurred significant interest in the development of advanced membranes for various separation processes. The development of efficient technologies for GHG separation and capture and water treatment are crucial for addressing these environmental issues.

The environmental strategies of the European Union and the USA state the intention of achieving carbon neutrality in 2050 by optimizing renewable energy sources and stimulating circular economy to prevent GHG emissions [4,5]. Also, regarding water scarcity, agriculture and industry are turning to seawater desalinization [6,7], among other separation and purification membrane-driven technologies. Industrial processes are very sensitive to changes in their operational parameters. Thus, the substitution of fossil fuels by cleaner sources of energy is not expected to happen quickly [8]. The most realistic option at industrial levels is to apply pre- and post- combustion treatments to either liquid or gas energy vectors to optimize the calorific value of the liquid or gas stream and enhance the elimination of atmospheric pollutants within flue gases. Also, several parameters are critical for the environmental welfare in wastewater treatment, such as the aeriation rate for activated sludge production, which controls emissions of GHGs to the atmosphere in this process [9]. 

In this context, membrane technology has risen as an emerging, less energy-intensive system for environmental purposes during the past years [6,7]. Polymer membranes can perform different green applications with a wide range of materials: polyolefins [10,11], fluoropolymers [12,13], polyesters [14,15], etc. Using membrane-driven technologies, several components can be recovered from the main effluent, whether in the gaseous or aqueous phase, such as biogas or syngas for their use as an energy vector at the industrial level [16] or, in the case of a liquid effluent, the recovery of substances of interest like phosphorus [17].

In the case of CO_2_ separation, CO_2_-capturing technologies have been researched thoroughly during the last decades, consisting mostly of absorption [18], but also adapting adsorption [19] and cryogenic distillation [20] for this purpose. Among these, absorption with amines [18,21] is the most popular technique because of its absorption effectiveness (0.6–0.7 moles CO_2_/mole amine [21]) and solid establishment and control in the industrial activity [22]. However, all these technologies have common drawbacks, namely their extremely high energy intensiveness, especially in the case of amine absorption (3.6–3.7 MJ/kgCO_2_), and their elevated capital and operation costs [21,23]. Cryogenic distillation also suffers from an elevated energy consumption (1.8–1.9 MJ/kgCO_2_), but it is still under development for CO_2_ and CH_4_ [20]. Membrane technologies are still not competitive in economic and technical terms [24] since amine absorption provides economic savings up to 13 USD/tCO_2_ [24]. 

However, polymer membranes in general may be competitive in these terms in the near future due to (i) a widening in the available membrane materials used for more specific environmental applications, including gas separations, pervaporation and, especially, treatment of liquid streams, from which components of interest such as metallic ions or phosphorus can be recovered [25,26,27,28] and (ii) their readiness to incorporate additives and other performance enhancers to help mitigate this viability gap. This is the case of deep eutectic solvents (DESs) [29,30], which are the focus of the present review.

Deep eutectic solvents (DESs) are substances of the family of neoteric solvents due to its sustainability and availability [31] and the versatility of their applications [32,33]. These substances consist of a mixture of one hydrogen bond acceptor (HBA) with at least one hydrogen bond donor (HBD), which are intimately associated by hydrogen bond interactions, subsequently decreasing the melting point of the DES. The physicochemical properties of DESs depend on the specific molar ratio of the mixture, apart from the intrinsic nature of the employed species, which offers a wide tunability range and performance. As a cornerstone example, the DES solubility of CO_2_ has been reported to be around 0.31 mole CO_2_ per mole of DES for the case of ChCl:Urea at a 1:2 molar proportion at a pressure of 12.5 MPa [34], which is a promising feature considering the novelty of their application for decarbonization separation membrane technologies, despite the extreme pressure conditions. Research is focusing on the use of DESs to functionalize polymer membranes to enhance their performance by means of different methodological approaches. Consequently, this review encompasses (i) the understanding of DES properties; (ii) the use of DESs as additives during the synthesis of polymer membranes as pore formers; and (iii) the functionalization of these membranes by DESs to obtain liquid membranes. Also, this review will propose new promising research challenges that might help foresee future milestones for the use of DESs in membrane technology.

## 2. Properties of Deep Eutectic Solvents

In this section, an overall vision of DESs is given, in terms of structure and properties. Some of the most common hydrogen bond donors (HBD) and acceptors (HBAs) used in the formulation of these eutectic mixtures are shown in Table 1. DESs have several characteristics that make them attractive for membrane design and modification, namely being difficult to volatilize and non-combustible while remaining in a liquid state at room temperature and also having overall good gas solubility [35]. Interestingly, under the bioeconomy framework, some natural plant metabolites like organic acids or nitrogen-based compounds may be employed for the elaboration of DESs, which receive the name of natural DESs or NADESs [36]. The feasibility of this type of substances results in a great availability of resources, with a very low cost and easy preparation [36]. In general terms, NADESs fulfill several of the “Twelve principles of the Green Chemistry” [37], mainly due to their sustainability, since they are renewable resources, plant-based compounds, or even waste materials from agriculture [38]. These features make DESs cost-effective, widely available, and decreases the dependence on fossil resources. They are less hazardous and intrinsically safer for both human health and the environment [39] since they are innocuous and their low vapor pressure offers less chemical exposure risk in both research and industrial environments. DESs offer a good biodegradability and biocompatibility [36], since DESs are often composed by materials that are assimilable by the environment [40,41]. All these features make DESs an attractive green alternative to traditional solvents.

### 2.1. Classification of Deep Eutectic Solvents

According to the literature, DESs have been traditionally classified into four types. However, a new fifth type has been introduced quite recently. All these DES types are described in Table 2. DES types I, II, and III use HBAs based on ionic compounds. Regarding HBDs, types I and II use different ret species (hydrated or not) as their HBDs, typically based on Fe, Cr, Ni, or Co. Type III, on the contrary employs HBDs based on organic compounds, such as amines, amides, or organic acids. On the same page, type IV uses the same HBDs, but uses metallic salts as HBDs. In contrast, type V DESs is characterized by not possessing ionic species. 

While certain non-ionic chemical mixtures have been described as DESs, the majority are just mixtures that adjust to thermodynamic ideality or have negative deviations from it. The actual key to producing non-ionic DESs, which can be categorized as type V, is assigned to be the acidity difference between functional groups of HBA and HBD [42]. Among these different groups, choline chloride (ChCl) has been the most studied HBA because of its high polarity [43], which increases the DESs capacity to solvate a great variety of chemical species. Additionally, the solvation capability of ChCl is favored by the forementioned hydrogen bond interactions. Hydrogen bonds are somehow strengthened by transition metals, metallic halides, and oxides, due to the intensity of the ion pairs [44].

### 2.2. Physicochemical Properties of Deep Eutectic Solvents

Table 3 compilates the following physicochemical properties of DESs with different HBA and HBD classified by types: (i) melting point, (ii) viscosity, (iii) surface tension, and (iv) density. An explanation of the influence of each factor is given in the following paragraphs.

#### 2.2.1. Melting Point of Deep Eutectic Solvents

**Figure 1:**
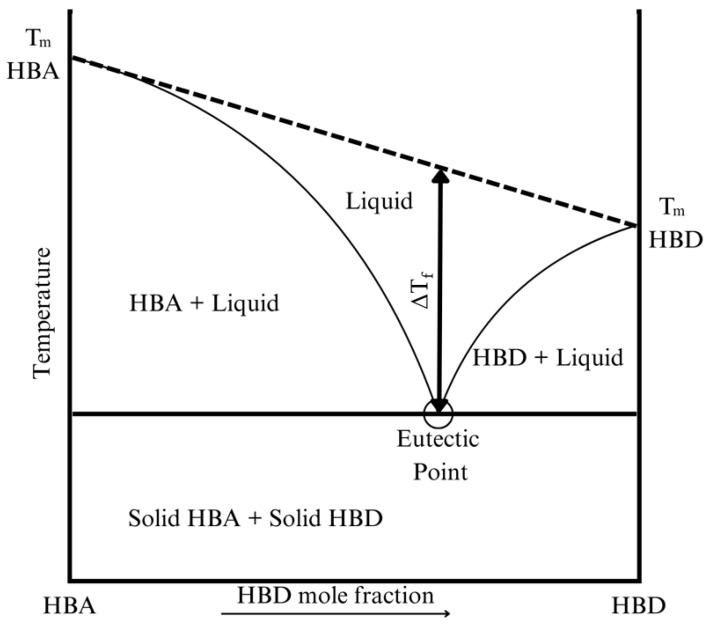
Typical phase diagram of a deep eutectic solvents at atmospheric pressure. T_m_ being the melting temperature and ΔT_f_ being the melting temperature depression of the deep eutectic solvent with respect to the theoretical melting point.

Regarding the melting point of DESs, the depression of this temperature is controlled by thermodynamic parameters, such as the lattice energy of the mixture, and the entropy changes through the process of forming a liquid phase [78,79]. A wide range of liquid state is critical for a DESs, since it reduces the sensitivity of the additive to temperature changes, which may provoke its solidification. The typical behavior of these eutectic mixtures is represented by the example shown in Figure 1.

To obtain an especially large melting point depression, HBDs with functional groups such as amide and halide groups are prone to further decrease the melting point of the mixture [80]. A common example of these functional groups is the combination of ChCl with Urea. Specifically, the mixture ChCl-Urea (1:2) forms a very stable liquid phase at room temperature, having a melting temperature of 285 K, by displaying different types of complexations between the Cl^-^ anion and the urea molecule [81,82].

Analogously, carboxylic groups found in organic acids tend to complexate the halide ion instead of dissociating into a proton and a carboxylate species [68]. DESs featuring carboxylic groups achieve a larger melting temperature depression than amide groups. However, since organic acids often have a higher fusion point than urea, the melting temperature of DESs with carboxylic groups remain higher than the latter ones [68].

A high number of hydrogen bond interactions offers a higher charge delocalization, which aids the melting temperature reduction [78,83]. Thus, DESs consisting of ChCl and D-Fructose (FR)—which has five hydroxyl groups that provide five hydrogen bonds—also has a low melting point of 283 K at a molar ratio of 2:1, respectively [64]. 

Regarding other DESs compositions, thymol-based and menthol-based DESs are an attractive combination, given their low melting temperature after a notable depression of the melting point of the pure species, which is particularly interesting due to the similarity of both molecules [84]. 

DESs containing amide or carboxylic functional groups are more prone to remain in a liquid phase either alone or introduced into a dope solution, due to the elevated melting temperature depression and avoiding the future alteration of the membrane homogeneity. Regarding the fabrication of polymer membranes, such as poly(vinylidene fluoride) (PVDF), adding DESs, including the mentioned groups, also enhances the formation of a porous polymeric matrix. Amide and carboxylic DESs enhance the pore forming process, since their high polarity hasten the migration of solvent out of the membrane [85]. This is also an advantage for membrane functionalization, since it is possible to form selective liquid membranes that have low sensitivity for changes in the ambient conditions [29,36]. All these aspects will be explained in detail in the corresponding chapter.

#### 2.2.2. Surface Tension of Deep Eutectic Solvents

Surface tension in DESs can be understood as a measure of the cohesion between the molecules of the solvent. Thus, surface tension quantifies the energy required to increase the surface of a fluid by a unit of area (mJ·m^−2^, or mN·m^−1^ in IS units) [67]. Temperature is one of the most affecting factors for this parameter since an increase in temperature directly implies a decrease in surface tension. This drop is due to intermolecular vibrations caused by the kinetic energy increase, which reduces the cohesion on the surface of the liquid, making the liquid surface more unstable [86]. Moreover, it also depends on the type of salt used and the HBA/HBD ratio [67]. 

Thus, an increase in the size or the amount of HBA used lowers the surface tension due to the reduction in these interactions [79,87]. The literature reports that DESs based on ChCl and organic acids such as lactic acid at molar ratio 1:2 have a notably low surface tension, with a value of 47.4 mN·m^−1^ at room temperature [88], descending to 44.4 mN·m^−1^ when the molar ratio of lactic acid is increased to 1:4. In contrast, intricate hydrogen bonding increases the surface tension values. An example of this is the surface tension of water, 72 mN·m^−1^ at room temperature, which has plenty of these hydrogen bond interactions [67]. Its influence is connected to that of the viscosity, which is shown in the next section.

#### 2.2.3. Viscosity of Deep Eutectic Solvents

Related with the mentioned ChCl-based DESs, these compounds are characterized by a high viscosity, which directly influences the mass and energy transfer capacity and also the operability of the resulting DESs [89]. 

At a microscopic level, viscosity is affected by the mobility of the DESs molecules. Regarding this, hole theory states that, on melting, DESs molecules create empty spaces. These spaces have a random ordination among the liquid phase and also have a variable radius, which may allow the movement of molecules if the size of the empty space is big enough. The average size of these space radiuses (*r*) can be obtained as a function of the previously described surface tension property (γ) following Equation (1) [90], where *k* is the Boltzmann constant and *T* is the temperature of the system.

(1) $$4\cdot \pi \cdot r^{2}=\frac{3.5\cdot k\cdot T}{\gamma }$$

For species that have a significant surface tension value, the empty size radius is prone to be smaller than another species with lower surface tension at the same temperature. Thus, as the empty space is smaller, the mobility of the molecules will be more difficult, increasing the viscosity value.

The hole theory can be demonstrated observing the behavior of DESs with the presence of carboxylic or hydroxyl functional groups in their HBDs, which lead to a direct increase in hydrogen bonds of the solvent and also translate as a rise in the viscosity of the DESs due to the lack of empty spaces of significant size for the movement of molecules [69].

A clear example is the mixture ChCl-Oxalic Acid at a 1:1 ratio, which has a viscosity value of 8953 mPa·s at room temperature (298.15 K) [69]. In contrast to this, it is reported that adding a salt like ChCl to glycerol decreases the viscosity of the latter. Thus, in a DESs consisting of ChCl and glycerol (GLY) at a 1:2 molar ratio, respectively, the viscosity value decreases to approximately 400 mPa·s versus the original glycerol viscosity (1200 mPa·s), and the viscosity still decreases as the molar ratio of the ChCl is augmented [60]. This phenomenon contrasts with ChCl–diol mixtures, where the viscosity of the DESs increases with the concentration of ChCl. An example of this behavior is the mixture ChCl:1,4-butanediol, which presents a viscosity value of 78 mPa·s at a ChCl concentration of 5%, which increases to 140 mPa·s when the ChCl concentration increases to 25% [91].

In any case, it also has been observed that the volumetric aspects, such as free volume fraction, are more influential than the interaction between HBA and HBD [87]. This can be clearly seen for other quaternary ammonium salts apart from ChCl, in which the alkylic chains attached to the molecule may provide different effects: in tetramethylammonium species ([N_1111_] ^+^), their short chains cannot completely delocalize the charges of the anion attached to this molecule, increasing the viscosity of any formed DESs. The same result is obtained with tetrabutylammonium molecules ([N_4444_] ^+^), but, in this case, the reason is excessive steric resistance since the contact area of the molecule is noticeably higher. An equilibrium between delocalization and avoidance of free volume is needed, and it may be found in tetraethylammonium molecules ([N_2222_] ^+^) [48]. 

Other solutions pass by the addition of water, which lowers the viscosity, but it also decreases the density of the solvent [69,92]. 

Regarding membrane design, viscosity is relevant in terms of controlling the polymer matrix fabrication. Either if DESs are used as pore-forming additives or co-solvents of the dope solution, viscosity, together with surface tension, govern the migration mechanisms of these additives from the membrane. Generally, higher viscosities and surface tension values induce a lower migration rate, as detailed in the specific chapter below.

#### 2.2.4. Density of Deep Eutectic Solvents

Lastly, density is also a relevant parameter for DESs for the design, operation, and optimization steps of their processing [87]. This feature is especially important in membrane functionalization applications where the available working volume is reduced. 

Following the forementioned hole theory, density varies from one DESs to another not only by the nature of its composition, but for the availability of empty spaces. Low surface tension species possess empty spaces with higher radius, diminishing the density of the DESs. This phenomenon establishes density as another variable for the control of membrane synthesis kinetics, similarly to viscosity and surface tension.

Thus, small functional groups and chains provoke better packing of the species and a consequent increase in surface tension, so groups such as hydroxyls contribute to increase the density of the developed DESs [69,93]. Thus, examples of high-density DESs may be based on glycerol (GLY) or sugar species—D-glucose or D-fructose (FR)—HBDs. DESs consisting of ChCl:D-glucose or ChCl:FR at a 1:1 molar ratio, possess a density of 1.272 and 1.273 g/cm^3^, respectively [63]. In contrast, aromatic groups provide DESs with more steric resistance, lowering the density of the mixture [71]. Hence, phenolic DESs will have less density than the forementioned DESs. Common examples of aromatic DESs are the ones consistent on phenolic compounds, such as phenol and o-cresol. ChCl:Phenol and ChCl:O-cresol at a molar ratio of 1:2 have densities of 1.097 and 1.078 g/cm^3^, respectively [71].

The application of DESs for the fabrication and functionalization of polymeric membranes is still a work in progress, but research is being conducted to improve the knowledge about the combination of these species as membrane synthesis enhancers either as pore formers [94,95], co-solvents for the polymer [96,97], as additives [98,99], or other functions in the synthesis process [100,101]. Also, regarding membrane functionalization, DESs are included for the formation of liquid membranes and especially supported liquid membranes (SLMs) [27,36,102,103]. These features will be discussed during this work.

## 3. Use of Deep Eutectic Solvents in Polymer Membrane Fabrication

The design of polymer-based membranes needs to ensure a controlled structure. Reverse osmosis (RO), microfiltration (MF), ultrafiltration (UF), or nanofiltration (NF) processes are usually performed by porous membranes, and therefore both porosity (ε) and pore size (r_p_) are critical parameters in their design. Also, solubility and solidification kinetics play an important role in the membrane structure. The prediction of solubility power of reported DESs indicates that their utilization in polymeric membrane fabrication seems to be a challenge since they have low δ_p_ and high δ_h_ solubility parameters compared to conventional and newly suggested solvents. Therefore, further studies for synthesizing different DESs with specific solubility parameters to dissolve each type of polymer or for optimizing the whole fabrication process are required [104]. This section covers the impact of different DESs on these features. 

Table 4 comprises a collection of works which include DESs during the fabrication of polymeric membranes of different natures. The main features of functionality are given in terms of r_p_, ε, and static water contact angle (WCA). The table is structured by the type of activity of the DESs and for different polymer families. References to specific studies are given in the following subsections hereinafter.

The effect of the DES addition into the dope solution in the final membrane properties can be different, relying on the nature of DESs, polymer, solvent, and the applied method. Hence, the type of DESs activity can be classified mainly in three categories: (i) additive and/or modifier when DESs is usually used at low concentrations < 5% for tuning the final membrane physical-chemical properties (addition of new functionalities, change in hydrophobicity, etc.) and controlling the membrane formation process (control of the polymerization kinetic, inhibition of reactions); (ii) pore former when DESs is mainly used for tailoring the porous structure of the membrane and DESs is not usually present in the final membrane structure; and (iii) solvent or cosolvent when DESs is usually used at concentrations > 50% in the dope solution to dissolve the base monomers and other additives.

One of the most traditionally used techniques for polymeric membrane fabrication in the literature is the non-solvent induced phase separation (NIPS). NIPS consists of a three-component system: the polymer, a solvent, and a non-solvent [105]. The polymer is responsible for the membrane structure formation and is immiscible with the non-solvent component, usually water. Also, there is a solvent species which dissolves the polymer and allows the membrane casting by making the polymer-based solution, also known as dope solution [106]. This way, the solvent and the pore formers leave the polymeric matrix due to the high affinity between these species with the non-solvent [106], decreasing the free energy of the cast polymer, with the further solidification of the polymer. Hence, the high affinity between the solvent and the component of the coagulation bath leads to the formation of empty voids within the polymeric structure [107,108,109]. Similar to NIPS, it is also easy to find works that use membranes prepared by phase inversion methods based on different driving forces in the literature, such as non-solvent induced phase separation with temperature gradient (NTIPS), which use temperature as a driving force to create the pores inside the semi-permeable structure [110], in addition to the chemical affinity between solvent and non-solvent species. Other fabrication techniques are casting evaporation (CE), interfacial polymerization (IP), co-solvent-assisted interfacial polymerization (CAIP), and electrospinning (ES). The CE method consists of the evaporation of the solvent contained in the casting solution to achieve the membrane formation. For the IP method, a suitable substrate is used, which allows the polymerization of the reagent to the membrane formation at the two-phase interface. Regarding the CAIP method, additives as co-solvents are added to the membranes prepared by IP during the reaction, with the aim of improving some of the characteristics of the membranes produced. Finally, the use of the ES technique for the preparation of membranes allows for unique scaffolding non-woven structures.

Examples of the most common solvents are n-methyl-pyrrolidone (NMP), dimethyl sulfoxide (DMSO), or dimethyl formamide (DMF) [111]. In addition, membrane solutions are commonly prepared including pore formers as additives to increase and tailor the porosity of the membrane. Glycerol (GLY), poly(ethylene glycol) (PEG), poly (vinyl pyrrolidone) (PVP), and LiCl are presented as conventional pore formers [112]. Since the negative environmental impact of this type of compound, the use of ionic liquids (ILs) and DESs as a pore former and/or solvent, has recently appeared as a promising approach to reduce the toxicity of the membrane preparation process [113]. However, this approach is at an early stage of development with few published research works, and the cost-effectiveness of a large-scale process has not been demonstrated as feasible yet [114].

The incorporation of DESs as additives, solvents/co-solvents, pore formers, and for other uses is offered in a compilation in Table 4 and Table 5, together with an indication on the main structural properties and an explanation of the main influence on the process conditions for several separation technologies. The following subsections dive deep into the main characteristics of each methodological approach.

**Table 4:** Compilation of the published works using deep eutectic solvents (DESs) for the preparation of flat-sheet polymeric membranes and the main properties of the obtained membranes. HBD: hydrogen bond donor; HBA: hydrogen bond acceptor; C_DES_: DES concentration in the dope solution; M.P.T.: membrane preparation technique with indication of the non-solvent used for NIPS method; r_p_: pore size; ε: overall porosity; WCA: static water contact angle; AC: acetone; AcA: acetic acid; CAIP: co-solvent-assisted interfacial polymerization; CE: casting evaporation; DMAc: Dimethyl acetamide; DMF: Dimethyl formamide; DMSO: Dimethyl sulfoxide; ES: electrospinning; EtOH: ethanol; Hx: n-hexane; IP: interfacial polymerization; IPA: isopropanol; NIPS: non-solvent induced phase separation; NMP: N-methyl-pyrrolidone; NTIPS: non-solvent induced phase separation with temperature gradient.

Work	Base/Support Material (Solvent)	DES Code (Molar Ratio)	HBA	HBD	Nature of DES	C_DES_, %	M.P.T. (Non-Solvent)	r_p_, nm	ε, %	WCA, °	Membrane Morphology
Use of DESs as additive and/or modifier											
[99]	PES (DMAc)	ChCl/EG (1:2)	Choline chloride	Ethylene glycol	Hydrophilic	0.5–4.0	NIPS (H_2_O)	3.4–12.1	48.4–64.0	42.3–48.2	Dense top layer and a finger-like structure in the bulk
[98]	PES (DMAc)	L-M/CSA (5:1)	L-Menthol	10-camphorsulfonic acid	Neutral	0.2–1.0	NIPS (H_2_O)	0.73–0.85	70.5–82.7	~66	Top dense layer, an intermediate finger-like layer and a sponge-like bottom
[115]	PVDF (AC)	[BMIM][Br]/DEG (2:1, 1:1, 1:2)	1-butyl-3-methyl-imidazolium bromide	Diethylene glycol	Hydrophilic	1.25–5.00	CE				Dense
[116]	PA (H_2_O/Hx)/PSf (DMF)	ChCl/Urea (1:2)	Choline chloride	Urea	Hydrophilic	1–10	IP			27.7–36.6	Dense top PA layer and sponge-like PSf support
[117]	PA (H_2_O/Hx)/PSf	Th/AA (1:1, 1:2, 1:3, 1:4)	Thymol	Acetic acid	Hydrophilic	0.0025–0.02	CAIP				Dense top PA layer and porous PSf support
[118]	PA (H_2_O/Hx)/PES	CA/ChCl/GLY (x:1:2, ternary DES with x ranging from 0.24 to 0.96 mol%)	Choline chloride	Glycerol	Hydrophilic	10	IP			47–64	Cellular/Nodular structure
			Citric acid	Citric acid							
[119]	PAN (DMF)	SAT/Urea (1:2)	Sodium acetate trihydrate	Urea	Hydrophilic	~3–~15	ES			50–70	Porous
[120]	PI (NMP + DMAc)	ChCl/EG (1:2)	Choline chloride	Ethylene glycol	Hydrophilic		NIPS (H_2_O + NMP)	135–152	42–67	58–74	Finger-like microporous
[121]	PAI (NMP)	ZnCl_2_/AA (1:3)	Zinc chloride	Acetamide	Hydrophilic	5–50	CE			65.3–84.1	Dense. Macrovoids appeared at DES concentrations of >20%
[122]	PEBAX (H_2_O + EtOH)	[EMIM][Cl]/EG (1:1)	1-ethyl-3-methylimidazolium chloride	Ethylene glycol	Hydrophilic	~5–~15	CE				Dense
		[EMIM][Cl]/Lev (1:2 1:1)	1-ethyl-3-methylimidazolium chloride	Levulinic acid	Hydrophilic						
[123]	CS-CMC (H_2_O + AcA)	ChCl/Urea (1:2)	Choline chloride	Urea	Hydrophilic		CE				Dense
[124]	CS (H_2_O + AcA)	PPRO/GLU (5:1)	Protonated L-proline	Glucose	Hydrophilic	5	CE			50	Dense
[125]	CS (H_2_O + AcA)	PCA/SULF (1:3)	Protonated 2-Pyrrolidone-5-carboxylic acid	Sulfolane	Hydrophilic	5	CE			100	Dense
[126]	CS (H_2_O + AcA)	PRO/SULF (1:2)	L-proline	Sulfolane	Hydrophilic	5	CE			34	Dense
[127]	GO (H_2_O)/PES	ChCl/EG (1:2)	Choline chloride	Ethylene glycol	Hydrophilic		CE			56.5–62.9	Porous
Use of DESs as pore former											
[128]	PES (NMP)	[N4444][Cl]/IM (3:7)	Tetrabutylammonium chloride	Imidazole	Hydrophilic	2	NIPS (H_2_O)	40.5 ± 1.2	86.5 ± 2.5		All: Dense top layer and a porous sublayer with a finger-like and macrovoid structure
		[N4444][Br]/IM (3:7)	Tetrabutylammonium bromide	Imidazole	Hydrophilic	2	NIPS (H_2_O)	32.2 ± 0.8	63.3 ± 1.8		
		[P4444][Cl]/IM (3:7)	Tetrabutylphosphonium chloride	Imidazole	Hydrophilic	2	NIPS (H_2_O)	37.1 ± 0.8	78.4 ± 1.9		
		[P4444][Br]/IM (3:7)	Tetrabutylphosphonium bromide	Imidazole	Hydrophilic	1–4	NIPS (H_2_O)	38.3 ± 1.0 (at 2%)	83.6 ± 2.0 (at 2%)		
		[BMIM][Cl]/IM (3:7)	1-butyl-3-methylimidazolium chloride	Imidazole	Hydrophilic	2	NIPS (H_2_O)	34.9 ± 0.6	68.1 ± 1.8		
[95]	PES (NMP)	DecA/[N_4444_][Cl] (2:1)	Tetrabutylammonium chloride	Decanoic acid	Hydrophobic	1–4	NIPS (H_2_O)	12.90–16.94	62.46–74.10		Dense top layer, intermediate finger-like sublayer, and macrovoids and sponge-like structure at the bottom.
[129]	PES (DMSO)	ChCl/Urea (1:2, 1:3, 1:4, 1:5)	Choline chloride	Urea	Hydrophilic	1	NIPS (H_2_O)	14.24–29.46	70–93		Porous
[130]	PES (NMP)	ChCl/IA (1:1)	Choline chloride	Itaconic acid	Hydrophilic	0.5–0.8	NIPS (H_2_O)	1.96–2.93	71.0–73.2	60.9–66.2	Dense top layer and a finger-like intermediate layer with or without macrovoids
[131]	PSE (DMF)	ZnCl_2_/EG (1:4)	Zinc chloride	Ethylene glycol	Hydrophilic	1–10	NIPS (H_2_O)	24.4–54.3	77–84		Dense top layer and a finger-like and/or macrovoids with a sponge-like structure in the bulk
[85]	PVDF (DMF)	ChCl/Urea (1:2)	Choline chloride	Urea	Hydrophilic	2	NIPS * NIPS * NIPS * NIPS * NIPS * * (H_2_O)	532 ± 152		~71	All: Thin porous layer and a finger-like with macrovoids bulk. Macrovoids diminished as DES% increased
		ChCl/GLY (1:2)	Choline chloride	Glycerol	Hydrophilic	2		326 ± 88		~67	
		ChCl/ZnCl_2_ (1:2)	Choline chloride	Zinc chloride	Hydrophilic	2		350 ± 67		~60	
		ChCl/LA (1:2)	Choline chloride	Lactic acid	Hydrophilic	2		296 ± 138		~62	
		ChCl/GLU (1:2)	Choline chloride	Glucose	Hydrophilic	2		282 ± 45		~75	
[132]	PSf (NMP)	ChCl/FR (1:1)	Choline chloride	D-(-)-Fructose	Hydrophilic/Hydrophobic	1–4	NIPS (H_2_O)	4.4–17.0	55–88	36–75	Dense top layer and a sponge, finger-like, and macrovoid sublayer
Use of DESs as solvent or cosolvent											
[96]	PVDF (DES)	[NMA]/AA (1.9:1)	N-methylacetamide	Acetamide	Hydrophilic	81–85	NTIPS * NTIPS * NTIPS * * (H_2_O)	0.53–4.64	80–90	55–60	All: dense top layer, intermediate finger-like sublayer and macrovoided sublayer
		[NMA]/NMU (4.1:1)	N-methylacetamide	N-methylurea	Hydrophilic	83		5.37	~90	~50	
		[NMA]/NN′-DMU (2.8:1)	N-methylacetamide	N,N′ -dimethylurea	Hydrophilic	83		3.20	~80	~70	
[97]	PVDF (DES + PolarClean/TEP)	PTSA/TBnA MsO (1:1)	Benzyl-trimethylammonium mesylate	p-toluensulphonic acid	Hydrophilic	60	NIPS (H_2_O/IPA)	80–150	82–85	104–110	Sponge- and finger-like with macrovoids
		PhAA/TMG (2:1)	Trimethyl glycine	Phenyl acetic acid	Hydrophobic	60	NIPS (H_2_O/IPA)	150–280	80–85	104–114	Spherulitic
	PAN (DES + DMSO)	GLYA/TMG (2:1) (+)CSA/SB3-MIM (1.5:1)	Trimethyl glycine (3-(1-methyl-1H-imidazole-3-ium-3 -yl) propane -1-sulfonate)	Glycolic acid (1S)-(+)-10- camphorsulfonic acid	Hydrophilic Hydrophilic	60	NIPS (H_2_O)	230 ± 10	86 ± 2	~43	Finger-like with macrovoids
		(+)CSA/SB3-4 (2:1)	3-(N,N-dimethybutylammonio) propane-1-sulfonate	(1S)-(+)-10- camphorsulfonic acid	Hydrophilic	60	NIPS (H_2_O)	60 ± 10	82 ± 2	~42	Dense top layer and finger-like bulk
[133]	PA (H_2_O +DES/Hx)/PES	ChCl/EG (1:2)	Choline chloride	Ethylene glycol	Hydrophilic	10–90	IP			49–67	Porous
[134]	Lignin (DES)	PA/Urea (2:1)	Urea	Propionic acid	Hydrophilic	78	NIPS (H_2_O)			66–76	Finger-like
Use of DESs as non-solvent or a component in the coagulation bath in NIPS											
[100]	PVDF (DMAc)	BET/LA (1:2)	Betaine	Lactic acid	Hydrophilic	1–20	NIPS (H_2_O)			60–80	Finger-like macrovoids
Use of DESP as additive and/or modifier											
[101]	PA (H_2_O/Hx)/PI (NMP)	β-CD/MA (1:5, 1:10)	β-Cyclodextrin	L-malic acid	Hydrophilic	100 (DESP used for coating)	IP		47–79	6–17	Dense top layer and a porous bulk
[135]	CS (H_2_O)	β-CD/LA (1:3–1:8)	β-Cyclodextrin	Lactic acid	Hydrophilic		CE				Change from dense at highly porous as DESP concentration increased

**Table 5:** Compilation of the published works using deep eutectic solvents (DESs) for the preparation of flat-sheet polymeric membranes, their reported mean roughness (R_a_) or root mean square roughness (R_q_), and their performance in application. For more details about membrane composition and properties, see Table 4. PWF: pure water flux; MF: microfiltration; UF: ultrafiltration; NF: nanofiltration; RO: reverse osmosis; FO: forward osmosis; GS: gas separation; PV: pervaporation.

Work	Base/ Support Material	DES Code	R_q_, nm	Application	Performance	Concluding Remarks
Use of DESs as additive and/or modifier						
[99]	PES	ChCl/EG	7.5–12.9	NF for dye separation	PWF up to 241.3 L m^2^ h^−1^ at 3 bar with a BSA rejection of 98.9% and RG19 dye removal of 99.2%	The use of DESs always increased the PWF, and the antifouling properties were improved due to a smoother surface. The maximum PWF was obtained when using a membrane containing a 2% of DES.
[98]	PES	L-M/CSA	1.7–9.54 *	NF for pharmaceutical separation	PWF up to 111.5 L m^2^ h^−1^ with a ceftriaxone and amoxicillin rejection of 99.6 and 99.2%, respectively	The optimum performance membrane characteristics were obtained when using a membrane containing 0.2% of DES. Antifouling properties were also improved due to a smoother surface and higher negative surface charge.
[115]	PVDF	[BMIM][Br]/DEG		GS for removal of SO_2_	SO_2_ permeability reached up to 17,480 Barrer (0.2 bar, 40 °C) and SO_2_/N_2_ and a ultrahigh SO_2_/CO_2_ selectivity of 3690 and 211, respectively.	The increase of DES content and [Bmim]-to-DEG ratio improved the SO_2_ permeability and SO_2_/N_2_ selectivity. The maximum performance was achieved with the membrane containing 50% of DES (molar ratio of 2:1) and the membrane performance was stable for at least 100h.
[116]	PA/PSf	ChCl/Urea	12.6–30.1	RO for water desalination	PWF up to 56.7 L m^2^ h^−1^ with a NaCl rejection of 96.4%	The membrane modified with 1% of DES showed the best performance. Antifouling properties specially increased at DES concentration < 5% due to a smoother surface and the negative charge induced by the DESs.
[117]	PA/PSf	Th/AA	49.54–70.00	RO for water treatment	PWF up to 80.39 L m^2^ h^−1^ with a NaCl rejection of 98% at the optimal DES dosage.	Optimal DES dosage and molar ratio of 0.0025% and 1:3. This membrane also presented the best long-term performance stability with a flux decline of 9% after 24 h. In addition, the membrane presented the highest fouling resistance, showing a 1.5% flux decline after 180 min in operation with a NaCl/humic acid-containing solution, which was attributed to the roughness, water contact angle and Zeta potential reduction. Moreover, the use of DESs increased the membrane resistance to chlorine agents, improving the chemical washing efficiency of these membranes.
[118]	PA/PES	CA/ChCl/GLY	29.7–44.2 *	NF for ground and drinking water treatment	PWF up to 39.5 L m^2^ h^−1^ bar^−1^ with a Na_2_SO_4_ rejection of 98.8%	Best membrane performance obtained for the DESs containing 0.72 mol% of citric acid. Antifouling properties also enhanced due to a higher hydrophilicity and electronegativity (lower zeta potential).
[119]	PAN	SAT/Urea		Not evaluated		
[120]	PI	ChCl/EG	33.8–118.9	UF for aqueous phenol removal	PWF up to 300 L m^2^ h^−1^ with a phenol removal efficiency of 96%	The use of DES-coated nanosilica as nanofillers enhanced the membrane pore structure (increased pore size) and chemistry (increased hydrophilicity) for phenol removal, being the 2% nanoparticles load the optimal value for maximizing membrane performance
[121]	PAI	ZnCl_2_/AA		PV for water/IPA separation	Total flux between 30 and ~110 g m^−2^ h^−1^ with a separation factor between 200 and 800 at optimal DES content for a water in feed from 5 to 20%.	Total flux and separation factor inversely proportional and highly dependent of DES content. Total flux increased but separation factor drastically decreased as DES content rose. Optimal DES content established at 10%.
				GS for O2/He/N2 separation	He, O_2_ and N_2_ permeabilities of 5.32, 0.27 and 0.017 Barrer, respectively, and O_2_/N_2_, He/O_2_, and He/N_2_ selectivity of 15.9, 19.7 and 0.33, respectively.	Permeability coefficient for all gases increased with increasing DES content in membranes. However, the He/N_2_ and He/O_2_ selectivity decreased whilst O_2_/N_2_ selectivity increased.
[122]	PEBAX	[EMIM][Cl]/EG		GS for natural gas desulfuration	H_2_S permeability up to 1928 Barrer, and H_2_S/CO_2_, and H_2_S/CH_4_ selectivity up to 14.35 and 242.0, respectively.	The inclusion of DESs in membrane involved DES-H_2_S interactions, which improved the H_2_S separation efficiency, making the [Emim]Cl/Lev DES the most efficient.
		[EMIM][Cl]/Lev				
[123]	CS-CMC	ChCl/Urea		Proton exchange membrane for fuel cells	Proton conductivity up to 1.57·10^−2^ S/cm	DESs acted as a plasticizer which increased the thermal degradation stability of the membrane and promoted the proton conductivity. Also, DESs led to a smoother and more homogeneous morphology.
[124]	CS	PPRO/GLU	3.0 ± 0.5	PV for ethanol dehydration	Total permeate flux of 0.242 and 0.389 kg m^−2^ h^−1^ and separation factor of 1425 and 831.7 at temperature of 20 and 50 °C, respectively	Incorporation of DESs in membrane preparation improved the mass transfer of water molecules respect to ethanol, thus enhancing pervaporation yield and permeation.
[125]	CS	PCA/SULF	23 ± 0.5	PV for ethanol dehydration	Total permeate flux of 0.3 and 0.449 kg m^−2^ h^−1^ and separation factor of 518 and374 at temperature of 20 and 50 °C, respectively	Incorporation of DESs reduced the membrane mass transfer resistance, contributing to the increase in the permeate flux. However, membrane selectivity decreased respect to the CS bare membrane.
[126]	CS	PRO/SULF		PV for MeOH-MTBE azeotropic mixture separation	At 45 °C: total flux of 73 kg m^−2^ h^−1^ and separation factor of 1 At 25 °C: total flux of 8 kg m^−2^ h^−1^ and separation factor of 35	Higher separation factors were obtained with the crosslinked CS membranes even though the total flux drastically decreased respect to the non-crosslinked membrane. DESs incorporation in the membrane led to a decreased in specific mechanical properties such as Young’s modulus and tensile strength due to plasticization.
[127]	GO/PES	ChCl/EG	60.2–60.7	NF for dye desalination	PWF up to 124.8 ± L m^2^ h^−1^ bar^−1^ with a high dye rejection: - 99.4 ± 0.8% for Congo Red - 99.3 ± 0.6% for Direct Red - 96.7 ± 1.2% for Methyl Blue - 98.5 ± 0.8% for Evan Blue	The use of DESs as additive considerably increased the PWF due to the lower wettability, the enlargement of nanochannels after the DES functionalization, and the reduced yet high negative surface charge, among other factors. Moreover, Na_2_SO_4_ salt rejection was low in around 5% while keeping high dye rejection. In addition, GO/DES membranes presented an enhanced antiadsorption of dye properties and a flux recovery of 74–100% after four filtration cycles.
Use of DESs as pore former						
[128]	PES	[N4444][Cl]/IM	9.5 ± 0.2	UF for water treatment	PWF up to 781 L m^2^ h^−1^ with a BSA rejection of 97.7% at 2 bars	DESs as pore former increased the PWF due to the formation of nanovoids and enlargement of membrane pores. The maximum PWF was obtained for the N_4444_Cl/IM DES. However, DES-based membranes presented lower elongation at break and tensile strength than the PES bare membrane.
		[N4444][Br]/IM	6.4 ± 0.4			
		[P4444][Cl]/IM	9.1 ± 0.4			
		[P4444][Br]/IM	9.2 ± 0.2			
		[BMIM][Cl]/IM	7.3 ± 0.2			
[125]	PES	DecA/[N_4444_][Cl]		UF for water treatment	PWF up to 142.84 L m^2^ h^−1^ with a pepsin, egg albumin and BSA rejection of 91.5, 97.3 and 99.0%, respectively, at 2 bar	DES addition as porogen improved the water flux and protein rejection ratio of the membrane. The maximum membrane performance was obtained when using a 2% DES concentration.
[129]	PES	ChCl/Urea		UF for dairy wastewater treatment	PWF up to 233.9 L m^2^ h^−1^ with a rejection rates of TSS, TDS, BOD, and COD were stated at about 90, 88, 93, and 97%, respectively.	DES-based membranes significantly increased the permeate flux due to the larger pores, being the maximum PWF for the ChCl:Urea at molar ratio of 1:4. However, DES-based membrane are prone to be fouled in a short-time period with a permeate flux reduction of ~35% in 6 h of operation.
[130]	PES	ChCl/IA	28.43–30.48	NF for anionic and cationic dye separation	PWF up to 257.14 L m^2^ h^−1^ bar^−1^ with a dye rejection: - ~85% for Congo Red - ~70% for Methyl Orange - ~90% for Malachite Green - ~90% for Methyl Violet	The PWF was improved due to the induced hydrophily and roughness by DESs. In addition, the use of DESs drastically decreased the Mg, Na and Ca salt rejection, which improved the membrane selectivity. DES-based membranes slightly enhanced the antifouling properties.
[131]	PSE	ZnCl_2_/EG	3.6–9.5 *	UF for water treatment	PWF of 212.3 L m^2^ h^−1^ with a BSA, HA and SA rejection of 96.4, 82.7 and 97.4%, respectively, at 1 bar for the optimal DES doping content of 3%	Increase in DES doping content increased pore size, leading to a higher PWF even though the rejection of BSA, HA and SA drastically declined. Thus, the optimal DES content was stablished at 3%. In addition, membranes tended to suffer from a more severe fouling at DES content > 3% due to a higher water flux and surface roughness.
[129]	PES	ChCl/Urea	28.393	MF for water treatment	PWF = 62 L m^2^ h^−1^; BSA rejection = 45%	DESs application improved membrane thermal stability, even though overall tensile strength of the membranes were decreased from 4.24 to 2.92 MPa. Among the five tested DESs, glycerol-based DESs yielded the best membrane parameters and performances, showing an increase in permeate flux and maintaining an acceptable BSA rejection. Almost all membranes presented higher antifouling properties than the pristine PVDF due to a higher hydrophilicity.
		ChCl/GLY	24.338		PWF = 52 L m^2^ h^−1^; BSA rejection = 65%	
		ChCl/ZnCl	21.832		PWF = 38 L m^2^ h^−1^; BSA rejection = 64%	
		ChCl/LA	28.958		PWF = 6 L m_2_ h^−1^; BSA rejection = 100%	
		ChCl/GLU	36.970		PWF = 19 L m^2^ h^−1^; BSA rejection = 100%	
[132]	PSf	ChCl/FR	15.748–25.955	UF for stormwater treatment	PWF up to 125 L m^2^ h^−1^ bar^−1^ at the optimal DES concentration of 3%.	The use of FR-based DESs allowed tailoring membrane surface properties and pore size by adjusting DES concentration. DES-based membranes presented improved mechanical properties, and remarkable antifouling properties.
Use of DESs as solvent or cosolvent						
[130]	PVDF	[NMA]/AA		NF for water treatment	PWF = 97 L m^2^ h^−1^; BSA rejection = 96%	[NMA]/AA is considered as the optimal DES, leading to a high PWF and BSA rejection due to the favorable combination of membrane pore size, porosity, hydrophilicity, morphology and low mass transfer resistance. The use of DESs as solvent could drastically reduce the membrane fabrication cost due to the simplicity and low cost of these DESs compared to the toxic conventional ones and even other green solvents.
		[NMA]/NMU			PWF = 112 L m^2^ h^−1^; BSA rejection = 85%	
		[NMA]/NN′-DMU			PWF = 60 L m^2^ h^−1^; BSA rejection = 95%	
[97]	PVDF	PTSA/TBnA MsO	16–26 *	UF for water treatment	PWF up to ~2300 L m^2^ h^−1^ bar^−1^ and MB+ rejection ~75%	Differences in membrane pore size governed the water permeance and rejection of methylene blue cation dye (MB^+^) of the DES-based membranes. Thus, large pore sizes led to a higher PWF and membranes with similar pore sizes presented similar PWF.
		PhAA/TMG	19–34 *		PWF up to 3243 L m^2^ h^−1^ bar^−1^	
	PAN	GLYA/TMG (+)CSA/SB3-MIM	33 *		PWF = 2479 L m^2^ h^−1^ bar^−1^; MB+ rejection = 69%	
		(+)CSA/SB3-4	16 *		PWF = 874 L m^2^ h^−1^ bar^−1^	
[133]	PA	ChCl/EG	23.6–40.9 *	NF for water treatment	PWF up to 43.3 L m^2^ h^−1^ bar^−1^ with a Na_2_SO_4_ rejection of 99.3% at the optimal DES concentration.	Optimal DES concentration was 60% in the solvent. PWF increased a 143% respect to the bare membrane without using DESs. In addition, PWF and salt rejection kept constant during 6 days, indicating that DES-based membrane was stable. Sulfated salts (Na_2_SO_4_, MgSO_4_) presented a very high rejection whilst chlorine salts (MgCl_2_, NaCl) rejection was < 50%. Zeta potential of membranes decreased with DES concentration, which could improve antifouling properties against organic matter.
[134]	Lignin	PA/Urea		UF for molecular separation in the pharmaceutical and chemical industries	At 22% lignin in dope solution: - PWF up to 0.5 L m^2^ h^−1^ bar^−1^ - Methanol permeance of 0.14 L m^2^ h^−1^ bar^−1^ with γ-cyclodextrin rejection of 90% - Acetone permeance of 0.02 L m^2^ h^−1^ bar^−1^	The best outcomes were observed for membranes prepared at 22% lignin dissolved in a DESs with a molar ratio of 2:1. These membranes were suitable for treating aqueous and organic solvents. Regarding the membrane stability, an initial methanol flux decline of ~50% was observed in the first 20 h, even though the flux kept stable up to nearly 150 h.
Use of DESs as non-solvent or a component in the coagulation bath in NIPS						
[136]	PVDF	BET/LA	52.6–119	Static adsorption for rare earth ions separation	Adsorption capacity up to 39.3, 40.2, and 45.9 mg g^−1^ for Nd, Sm and Dy, respectively.	DES concentration of 5% led to the highest adsorption capacity which was higher than the bare membrane. DES addition in coagulation bath promoted the migration of hydrophilic functional groups in the membrane to lower epidermal layer, improving the adsorption capacity.
				MF/UF for rare earth ions separation	PWF up to 400 L m^2^ h−1 at 1% DES. Initial membrane flux up to 4.5 µmol m^−2^ s^−1^ at the optimal DES concentration.	PWF decreased as DES concentration increased due to a lower wettability behavior. Optimal DES concentration of 5%. Membrane kept the 70% of its initial flux after 7 operation cycles using EDTA as stripping solution and water for membrane flushing at the end of each cycle.
Use of DESP as additive and/or modifier						
[101]	PA/PI	β-CD/MA	42–109 *	FO for recovery of organic solvents	EtOH flux up to ~10 L m^2^ h^−1^ with a monascorubrin rejection of ~98%.	Solvent flux and monascorubrin rejection were significantly enhanced after the incorporation of a DESs interlayer. These effects were attributed to the large amount of hydrogen bonding induced by the DESs, and the β-cyclodextrin forming the interlayer has a cavity structure to prevent monascorubrin from penetrating through the composite membrane. The stability of the membrane with DESs interlayer was enhanced, showing an EtOH flux of ~7.5 L m^2^ h^−1^ after 600 min of operation. Flux decline was attributed to pore blocking, fouling, and swelling which destroy the membrane structure.
[135]	CS	β-CD/LA		Adsorption of dyes	Maximum adsorbent capacity for methyl orange of 203.5 mg g^−1^ at the optimal conditions with a removal efficiency up to 93%.	The highest adsorption efficiency was obtained at a DESs molar ratio of 1:4, 15 mL of PEG as pore former, dye solution pH of 5, methyl orange concentration of 20 mg L^−1^, and an adsorbent dose of 2 mg. Adsorption of methyl orange in DES-based membranes was favored by a higher influence of electrostatic interactions.

* Reported value of mean roughness (Ra).

### 3.1. Deep Eutectic Solvents as Components or Additives in Membrane Preparation

The use of DES as components or additives in membrane preparation has employed different types of polymers as base materials, meaning for that the polymeric material of which the final membrane will be made, materials such as polysulfone (PSF), polyamide (PA), chitosan (CS), poly(amide-imide) (PAI), poly(vinylidene fluoride) (PVDF), or polyethersulfone (PES), among others, are included. Some of the main applications studied in different works and presented in this subsection are listed as follows: reverse osmosis (RO); gas separation (GS); pervaporation (PV); microfiltration (MF); ultrafiltration (UF); and nanofiltration (NF).

**Figure 2:**
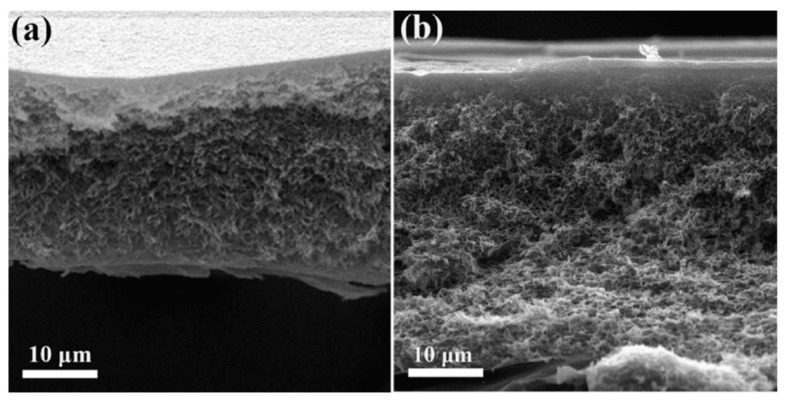
Cross-section by Scanning Electron Microscopy (SEM) images of the (**a**) unmodified and (**b**) modified membranes with 1 wt% of DES. Adapted from [116].

The inclusion of DESs as components or additives in membrane formation has been studied for different types of applications. One of the applications where DESs have been introduced is RO. Shahabi et al. [116] studied the use of the choline chloride-urea DESs in thin film composite (TFC) membranes, fabricated with PSF and PA by NIPS and IP. The immersion of membranes in DESs at a concentration from 1 to 10 wt% showed an unmodified surface, with only a decrease in roughness obtained with DESs inclusion. The cross-section of membranes presented a PSF support layer with a sponge-like structure and a PA top selective layer, as observed in Figure 2. The membrane with 1 wt% in DES showed the maximum pure water flux (PWF), with 56.7 L m^−2^ h^−1^.

Also, the effect of including DESs as an additive was studied for adsorption applications. Zhu et al. [135] prepared CS-based films with the addition of the DESs formed by β-cyclodextrin (β-CD) and DL-lactic acid (LA). SEM images showed how the DESs served as a plasticizer, increasing the roughness of films. The addition of DESs improved the removal of methyl orange-containing wastewater compared to the pure CS membrane, with a maximum of 92.79% when the ratio of CS to β-CD was 1:4.

Chitosan-carboxymethylcellulose membranes were prepared by Wong et al. [123] to study proton conduction and thermal properties. For that, DESs was added formed by ChCl and urea in the CE method for the membrane formation. A smoother and more homogeneous structure was obtained with DESs inclusion. It was shown that 50 wt% CS blend film, with the incorporation of DESs, was shown to be the highest in proton conductivity, 1.57 × 10^−2^ S/cm.

Pulyalina et al. [121], for GS applications, fabricated composites based on PAI and DESs. The DES was composed of ZnCl_2_ and acetamide in a molar ratio of 1:3, studying the influence of DES addition for concentrations between 5 and 50%. No apparent defects were found for the composites, and pure PAI showed a fully dense homogeneous structure. Above 20%, homogenous and no DES clusters were observed in the membrane. With the increase in DES content up to 50%, DES clusters were shown in the cross-sectional micrograph. The introduction of up to 20 wt% DES content led to a noticeable increase in the total flux, with a decrease in the separation factor when DES content increased. The membrane with 50 wt% DES concentration had the highest permeability, but the separation selectivity was dramatically lower.

**Figure 3:**
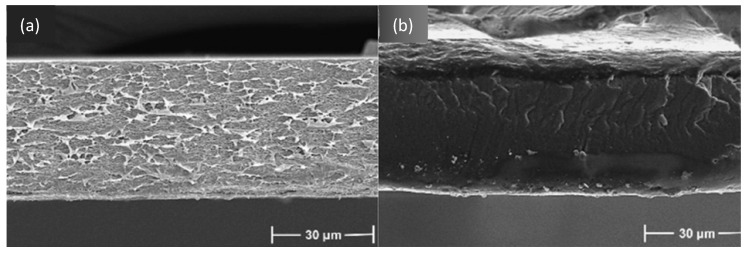
Morphologies of the prepared membranes. (**a**) Cross-section SEM images of neat PVDF membrane and (**b**) PVDF-DES (1:1) membrane. Adapted from [115].

Also, for gas separation, Zhang et al. [115] used the DESs formed by 1-butyl-3-metyl-imidazolium bromide ([BMIM][Br]) and diethylene glycol as an additive in a PVDF membrane, from 1.25 to 5.00 wt% concentration. For membrane formation, the CE method was used. DESs at different molar ratios between components and concentrations in the membrane were studied for that work. PVDF and PVDF-DES membranes images, which are included in Figure 3, showed a dense and homogeneous structure. The addition of DESs significantly increased the permeability and selectivity of the membranes for applications with SO_2_, achieving values of 17,480 Barrer in SO_2_ permeability and selectivities of 3690 and 220 for SO_2_/N_2_ and SO_2_/CO_2_, respectively.

Using PEBAX/DES blended membranes, Tu et al. [122] used 1-ethyl-3-methyllimidazolium chloride ([Emim]Cl)-based DESs for GS. Ethylene glycol (EG) and acidic levulinic acid (Lev) were chosen as HBDs to form DESs and were applied with a range of concentration from approximately 5 to 15 wt%. The results showed a more amorphous and wrinkled structure when [Emim]Cl-Lev was included, showing a dense and smooth morphology before that. The increase in DESs content and the use of Lev improved the permeability of H_2_S and the H_2_S/CO_2_-CH_4_ selectivity.

In addition, DESs have been studied in PV separation for different mixtures. Castro-Muñoz et al. [124] prepared CS membranes using the CE method and protonated-L-proline:glucose 5:1 as DESs. Membranes without and with 5 wt% of DES showed a smooth surface. The pristine membrane cross-section showed a dense crater-like structure, while the DESs generated a compact but less smooth morphology. DES inclusion increased the separation factor and permeation fluxes of water. Water/EtOH selectivity reached values up to 1427.

Also, Castro-Muñoz et al. [126], in subsequent studies, applied a 5 wt% concentration of L-proline: sulfolane (SULF) as a DES additive. CS membranes were prepared with the CE method, where all resulting membranes showed a smooth and uniform surface. The cross-section of membranes displayed a dense structure, while the addition of DESs was evidenced to provide a tighter and smoother structure. The selectivity in the methanol–methyl tert-butyl ether mixture was improved with DES incorporation and cross-linking up to 35.

For PV, Castro-Muñoz et al. [125] prepared CS membranes by the CE method, adding the DES 2-Pyrrolidone-5-carboxylic acid:SULF at a 1:3 molar ratio for 5 wt% concentration. Both DES-free and DES-containing flat membranes showed a defect-free surface but with an increase in roughness for DES addition. In SEM cross-section images no evidence was observed of pores or pinholes generation for DES addition. The membrane with DESs presented an increase in permeated flux and a separation factor of 518 for water/EtOH mixtures.

**Figure 4:**
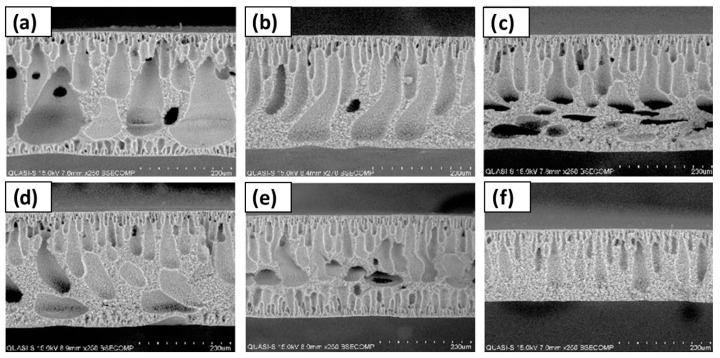
Cross-sectional SEM images of (**a**) PVDF, (**b**) PVDF-Urea-DES, (**c**) PVDF-GLY-DES, (**d**) PVDF-ZnCl_2_-DES, (**e**) PVDF-LA-DES, and (**f**) PVDF-Glucose-DES membranes. Adapted from [85].

For applications in MF, Yeow et al. [85] studied the use of five different DESs based on ChCl as the HBA. Urea, GLY, ZnCl_2_, LA, and glucose were the HBDs chosen for the study, all of them at a HBA:HBD molar ratio of 1:2 and 2 wt% concentration. PVDF was the membrane-former polymer, and the NIPS method was employed. Both pristine membranes and those with DESs showed homogeneous and microporous structures. As can be observed in Figure 4, an asymmetric morphology was observed for the pristine membrane with a porous top layer, a large macrovoid structure at the middle, and a suppressed finger-like structure at the bottom. The addition of DESs affected the viscosity of solution. The use of DESs with low viscosities, between 1 and 750 cP at 25 °C, showed an increase in the number of voids and enlarged the size in the bottom layer. Probably, since the bottom layer was not in contact with the coagulation bath, that structure could have appeared due to accidental separation of the forming membrane from the substrate. Having regard to the above, the viscosity of the dope solution plays a vital role in determining the membrane morphology, which affects the rate of solvent-nonsolvent exchange during the phase inversion process. Also, the incorporation of DESs increased the overall roughness and porosity surface, obtaining r_p_ from 0.28 to 0.53 µm with each one of the DESs analyzed. The PWF values of DESs with urea and GLY improved the obtained with the pristine membrane, reaching DES values of 62.49 and 51.90 L m^−2^ h^−1^, respectively.

**Figure 5:**
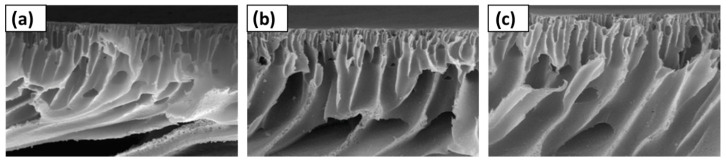
Cross-sectional SEM images of the prepared PES membrane. (**a**) PES/PVP 1wt%, (**b**) PES/PVP 1 wt%/DES 2 wt%, and (**c**) PES/PVP wt%/DES 4 wt%. Adapted from [99].

UF membranes were another application where Vatanpour et al. [99] incorporated a DES as an additive. PES membranes were fabricated using the NIPS method, incorporating PVP and ChCl:EG (1:2) (ethaline) DESs. The content in DESs was incorporated up to a 4 wt%, while the concentration in PVP was kept at 1 wt%. Figure 5 shows that all membranes presented an asymmetric structure with a dense upper layer followed by a finger-like porous bottom layer, indicating that ethaline did not alter the morphology of PES membranes. However, different concentrations of ethaline with PVP presence generated increases in the width of finger-like, and macrovoids were enlarged. In addition, DESs blended membranes demonstrated higher ε when ethaline concentration was increased up to 2 wt%. DES concentrations over 2 wt% increased the viscosity of the solution, quenching the creation of macrovoids and decreasing the membrane porosity (ε). The ε increased from 58.3 to 64.0% when the ethaline concentration in the PES/PVP solution incremented from 0 to 2 wt%. A similar trend was observed for r_p_ in the membrane, increasing from 8.5 to 12.1 nm in previous concentrations of ethaline. Again, the membrane at 2 wt% concentration in ethaline obtained the highest results for PWF, increasing up to 241.3 L m^−2^ h^−1^ at the transmembrane pressure of 3 bar.

Also, for UF, Ali et al. [120] prepared polyimide membranes embedded with silica-based nanofillers and coated with ethaline by post-impregnation by the NIPS method at 0.5–2.5 wt% (DESs-SiO_2_). The dispersion of DESs-SiO_2_ on the top surface of membranes caused a significant reduction in the width of the macropore channels and increased its density distribution. A slower demixing rate in the DESs-SiO_2_ coated membranes with the hydro-organic coagulation bath was attributed to an increase in the polarity of the casting mixture. Larger surface roughness was observed for coated DESs-SiO_2_ membranes, along with significantly larger estimated apparent porosity values than without ethaline addition. The largest PWF values, 300 L m^−2^ h^−1^, were obtained at the optimal 2 wt% nanofiller loading in the casting mixture. Also, the resulting DESs-SiO_2_-based membrane exhibited a maximum phenol removal efficiency of 96%.

Where most references to the use of DESs as membrane additives have been found was in NF, Mehrabi et al. [127] used ethaline as a novel DES for the functionalization of graphene oxide membranes produced by the CE method. A smoother surface was obtained in membranes with ethaline functionalization. These physical changes in graphene oxide membranes by DESs could increase the nanochannel’s size to facilitate the passage of water and improve the water permeance. However, an increase in the loading functionalization with the DES of membranes generated increments in membrane thickness, decreasing the mass transfer and water permeance. The highest water permeance obtained for the functionalized membrane was 124.8 L m^−2^ h^−1^ bar^−1^. 

Moradi et al. [98] used the L-menthol:10-camphorsulfonic acid DESs, at a molar ratio of 5:1, as a modifier of PES nanofiltration (NF) membranes. The NIPS method was used for membrane formation and DES concentrations varying from 0.2 to 1.0 wt%. When the DES concentration was 0.2 wt%, the maximum pore size (r_p_) was obtained, increased from 0.70 to 0.85 nm, and compared with membrane without additives. All fabricated membranes had a dense barrier on the surface, an intermediate layer with a finger-like structure, and a sponge-like bottom morphology. Regarding overall porosity, all modified membranes increased their porosity, reaching the maximum at 0.2 wt% DES content, with 82.7%. The reason for the increase in viscosity could be due to the lower viscosity for a concentration of 0.2 wt%, generating a higher mass transfer rate. All modified membranes showed lower surface roughness than the pristine membrane. The same dope solution that generated the membrane with the highest ε and r_p_ and 0.2 wt% DES concentration showed the maximum PWF with 111.5 L m^−2^ h^−1^.

**Figure 6:**
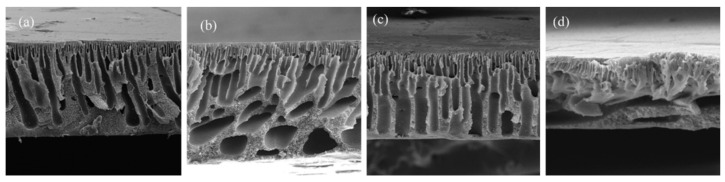
Cross-section SEM images of (**a**) neat PSF, (**b**) PSF/PVP 0.8 wt%, (**c**) PSF/DES 0.5 wt%, and (**d**) PSF/DES 0.8 wt%. Adapted from [130].

Also, for NF applications, Saeb el. al. [130] used a DES based on itaconic acid and ChCl as an additive for PSF-based membranes. The DES concentrations studied were 0.5, and 0.8 wt%, showing an asymmetric structure in all membranes. The hydrophilic nature of the DESs caused a faster transition between the solvent and non-solvent, forming larger pore channels, as shown in Figure 6. The number of finger-like pores in the membrane augmented with the increase in DES content and its r_p_ by connecting them, with values from 0.84 nm in the membrane without DES to 2.93 nm for 0.8 wt% in DES. In addition, the increase in the speed of the phase inversion process was the cause of the formation of larger pores and the development of macrovoids in the support layer, increasing the porosity ε from 59.44 to 73.21% in the membrane without and with the maximum concentration of DES, respectively. Moreover, the roughness of membranes was increased with the addition of DESs, due to the reaction of the hydroxyl functional group with the membrane surface. The water flux was enhanced by increasing the DES additive due to the higher hydrophilicity and surface roughness and the increase in r_p_ and ε of the membrane, enhancing a value close to 290 L m^−2^ h^−1^ for the membrane with a 0.8 wt% DES concentration.

To conclude with NF applications for DESs as an additive, Hao et al. [118] presented a study for PES and PA NF membranes fabricated by the IP method. A ternary DES was formed by different contents of citric acid (CA) and ethaline DES. The ternary DES concentration was 10 wt%, changing the CA content in the DES from 0 to 0.96 mol%. The increase in CA content made ternary DESs play an important role in forming a thinner PA separation layer. In addition, when CA content increased to 0.72 and 0.96 mol%, a cellular-like structure appeared on the surface, similar to the PES substrate. The optimum CA addition was determined to be 0.72 mol%, where the membrane showed a PWF of 39.5 L m^−2^ h^−1^ and a Na_2_SO_4_ rejection of 98.8%.

Regarding membranes fabricated by ES, Cui et al. [119] used polyacrylonitrile mixed with the DESs formed by sodium acetate trihydrate and urea, and gelatine at different concentrations. The gelatine/DES mixtures were studied at 5, 10, and 20 wt% contents. Uniform and bead-free nanofibers were obtained, whose diameter became smaller and displayed a more concentrated distribution in the presence of gelatine/DES.

### 3.2. Deep Eutectic Solvents as Solvent or Co-Solvent in Membrane Preparation

Urea-based, acetamide-based, ethaline, or different NADESs have been the DESs chosen as solvents or co-solvents in some works below. In addition to the NIPS method being the most employed method for membrane preparation with this new use of DESs, the most studied applications also coincide with those seen above. Micro- (MF), ultra- (UF) and nanofiltration (NF) applications stand out together with reverse osmosis (RO). A co-solvent is understood as a substance that modifies the properties (such as viscosity or kinetics of the NIPS method) of the solvent, up to a concentration of 50 wt/vol%. For RO, Dehqan et al. [117] used the NADESs formed by thymol and acetic acid at different molar ratios, 1:1, 1:2, 1:3, and 1:4, for each component as co-solvents. Polysulfone (PSF) was the support layer employed with a polyamide (PA) selective layer, where the organic phase for which preparation contained different amounts of NADESs. The content of the NADES at a molar ratio of 1:1 studied for the organic phase preparation were from 0 to 0.02 wt%. The membranes fabricated by interfacial polymerization (IP) or the co-solvent assisted interfacial polymerization (CAIP) method showed typical ridge-valley and leaf-like surface structures. When a concentration of 0.0025 wt% of NADES was used, it formed an island-like structure on the membrane surface, increasing its number with the NADES concentration. The thickness of these structures was reduced with the enhancement of NADES dosage. The PA membrane made by the IP method offered the highest surface roughness. Both IP and CAIP processes formed membranes with a very dense and non-porous selective layer, followed by a spongy PSF membrane. By the enhancement dosage of the NADESs to 0.01 and 0.02 wt%, the selective layer of the CAIP membrane became thicker. The border of the selective layer in the membranes with previous 0.01 and 0.02 wt% concentrations faded, and the selective layer transformed from a dense to a sponge-like structure. In terms of implementation, it was concluded that the CAIP process is a better method for creating RO membranes than the IP method, but the amount of the co-solvent must be optimized. After 7.5 h, the membrane fabricated with the NADESs at a 1:3 molar ratio and a very low 0.0025 wt% concentration presented the best performance with 71.50 L m^−2^ h^−1^ flux.

**Figure 7:**
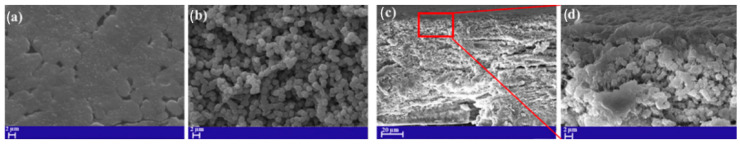
Scanning Electron Microscopy (SEM) pictures of PVDF membrane prepared with DES phenyl acetic acid/trimethyl glycine as a solvent and TEP as a co-solvent: (**a**) top surface, (**b**) bottom surface; (**c**) cross-section, magnified in (**d**). Reprinted from [97].

Russo et al. [97] studied the performance of poly(vinylidene fluoride) (PVDF) and polyacrylonitrile (PAN) membranes with DESs as solvents in ultrafiltration (UF). The types of DESs and NADESs used were halogen-free Brønsted acidic DESs, halogen-free zwitterionic carboxybetaine-based NADESs, and halogen-free zwitterionic sulfobetaine-based DESs. The NIPS method was employed for membrane preparation, using mixtures of DESs and Rhodiasolv^®^ PolarClean, triethyl phosphate (TEP), and DMSO as a co-solvent, with a molar ratio of 2:1. Different structures were obtained with each of the DESs employed. As presented in Figure 7, the membrane with TEP and the NADES formed by phenylacetic acid and trimethyl glycine was the membrane with the maximum mean flow pore size (r_p_), with 0.28 µm. Its structure presented a top porous layer and a spherulitic structure along the cross-section, suggesting a slow mass transfer, resulting in a particulate structure [136]. Also, this membrane showed the highest value for pure water permeability, 3243 L m^−2^ h^−1^ bar^−1^. Another cross-section structure that was observed in the membranes was the finger-like structure, obtained when mixtures of halogen-free zwitterionic sulfobetaine-based DESs and DMSO were used. Finally, a sponge-like structure was seen when isopropanol was added in the coagulation bath. In general, the porosity was quite similar for all membranes.

Nanofiltration (NF) applications have been studied, using different DESs as solvents and co-solvents. Fang et al. [133] prepared PA membranes by the non-solvent induced phase separation (NIPS) method using ethaline up to a 90 wt% concentration. Constructing a thinner PA layer is beneficial to inhibit the diffusion rate of the piperazine added to the dope solution via hydrogen bonding by the addition of DESs and increasing its concentration. In the membrane without the use of DESs, the surface showed a nodular structure compared with the PES substrate, with the structure disappearing with the increase in DES concentration. The membrane with 60 wt% concentration showed a cellular-like structure on its surface, associated with the thin thickness of the PA separation layer. SEM showed how the thickness decreased with the increase in DES concentration from 93 to 31 nm. The introduction of DESs improved the pure water flux (PWF) up to 43.3 L m^−2^ h^−1^ when the DES concentration was 60 wt%, and the Na_2_SO_4_ rejection achieved 99.3%.

**Figure 8:**
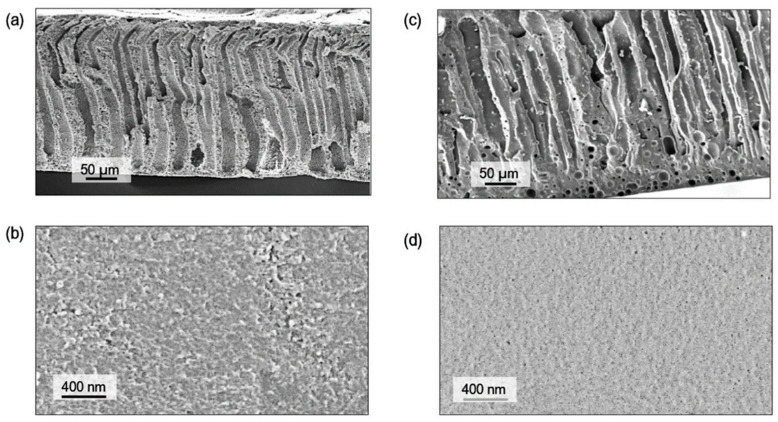
Cross-section (**a**,**c**) and surface (**b**,**d**) SEM images of membranes cast from 22 wt% lignin solution in DESs (**a**,**b**) before and (**c**,**d**) after crosslinking with 5% 1,4-butanediol diglycidyl ether in water. Adapted from [134].

For the two above mentioned applications, UF and NF, Gebreyohannes et al. [134] prepared lignin-based membranes with propionic acid and urea at a 2:1 ratio as the solvent. The method used for membrane formation was the NIPS process. The acidic nature and rich hydrogen bonding promoted a fast dissolution of the lignin at 80 °C. Figure 8 shows that asymmetric porous morphology was obtained before crosslinking, frequently observed for NIPS membranes. The addition of a DESs with low viscosity and high affinity for water contributed to a fast solvent/non-solvent exchange during the membrane formation, creating a highly finger-like porous structure. After crosslinking, a clear densification in the structure was shown with a more pronounced effect on the surface. The work concluded that the DESs could be used as a solvent without the addition of a co-solvent, and the cross-linked membrane had a water permeance of 0.5 L m^−2^ h^−1^ bar^−1^.

**Figure 9:**
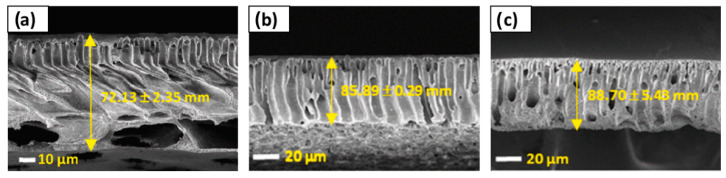
SEM images showing the cross-section, top surface, and bottom surface of the PVDF membranes prepared with different PVP concentrations and different DESs. (**a**) PVDF/PVP 2 wt%, (**b**) PVDF/PVP 2 wt%/NMU-DES, and (**c**) PVDF/PVP 2 wt%/NNDMU-DES. Adapted from [96].

Without the focus on any specific application, Ismail et al. [96] used another set of DESs dedicated to being used as solvents for PVDF membranes. Three DESs composed of n-methyl acetamide (NMA), which may be paired with acetamide (AA), n-methyl urea (NMU), and n,n-dimethyl urea (NNDMU), were used. The morphology of the obtained membranes using the different DESs as solvents was also affected at the work concentration, from 0 to 4 wt%. The membrane with AA-based DESs showed large macrovoids in the bottom layer of the membrane and a finger-like structure on top, as observed in Figure 9. On the contrary, the membrane with NNDMU-based DESs showed a sponge-like bottom layer structure. Finally, a finger-like structure over the whole membrane section was observed when NMU-based DESs was used. The maximum r_p_ obtained was 5.37 nm for 2 wt% concentration of NMU-based DES membrane. That membrane showed [96] a smaller bovine serum albumin separation factor but the highest PWF, 112 L m^−2^ h^−1^ bar^−1^.

### 3.3. Deep Eutectic Solvents as Pore Formers in Membrane Preparation

As previously introduced, both ε and r_p_ of polymer membranes are strongly dependent on the operational conditions of the dope solution casting during membrane fabrication, which affects the arrangement of the polymer chains during membrane formation, controlled by the solidification kinetics of the polymer in the corresponding non-solvent. Moreover, these parameters can be tailored by means of the use of different types of additives, commonly referred to as pore formers [137]. These pore formers can have either a solvating or non-solvating effect when incorporated into the membrane structure to create empty spaces or voids within the membrane [137]. Moreover, low porosity is reported to provoke more fouling issues due to the need of increasing the feed to the membrane [138]. Different compounds, normally called pore formers, have been commonly applied to preserve the porous structure of supporting membranes or modify the ε and r_p_ of the membrane, such as GLY or PEG [139,140]. Although the function of these compounds is generally to prevent the collapse of the porous structure of the membrane and not the direct creation of pores, they are called pore formers, which can lead to confusion regarding the functioning of these compounds. Therefore, a dissimilar term such as “pore retainer” could be more appropriate. In any case, the term “pore former” will be used, in order to continue prevent the reader from confusion with previous scientific reports. The most interesting type of pore formers reviewed in this work are those with a solvating nature, since they lead to the formation of micropores (diameter lower than 0.002 µm), interesting for gas–liquid separations, instead of mesopores (diameter from 0.002 to 0.05 µm) and macropores (diameter bigger than 0.05 µm) obtained by non-solvating pore formers [137]. In this subsection, studies about the use of DESs as pore formers with polymer substrates of PES, PSE, and PSF, for ultrafiltration purposes, are gathered.

**Figure 10:**
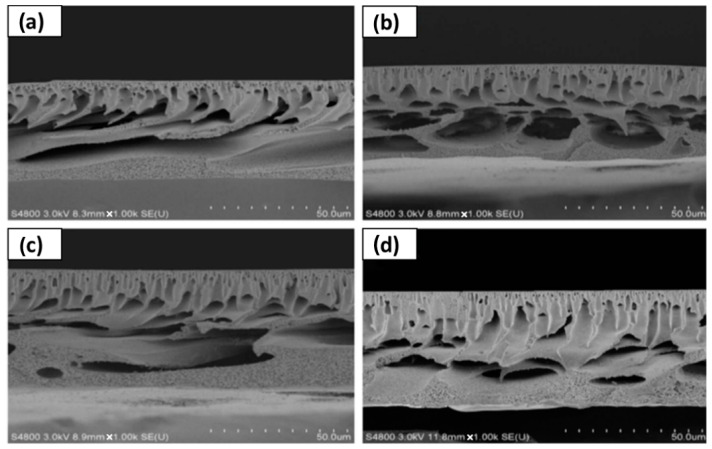
SEM cross-section images of the prepared membrane: (**a**) PES, (**b**) PES/DES 2 wt%, (**c**) PES/N_4444_Cl^−^ 2 wt%, and (**d**) PES/DecA 2 wt%. Adapted from [95].

Jiang et al. [95] used the DESs formed by decanoic acid (DecA), which acts as an HBD, and tetrabutylammonium chloride ([N_4444_][Cl]) as the HBA at a 2:1 molar ratio, respectively. PES was used as the former polymer for membrane preparation and the NIPS method for membrane formation. The collected results in Figure 10 showed a relatively uniform distribution along the membrane surface with a pore size between 12.90 and 16.94 nm, for 1 to 4 wt% DES concentration, respectively. Such behavior could be generated due to the thermodynamic instability of the casting solution induced by the addition of DESs. The overall porosity (ε) achieved its maximum when the DES concentration was 2 wt%, with a value of 74.10%, accepting this concentration as the optimum. In the case of SEM images, the membrane’s cross-section showed a typical asymmetric structure, with a dense skin layer, a figure-like intermediate layer, and a sponge-like bottom structure. Larger macrovoids increased with DES addition, decreasing the bottom sponge-like structure. Such changes in the membrane morphology could be interpreted by the phase separation mechanism during immersion precipitation, playing opposing roles in the instability caused by the DES addition and its viscosity increase at the casting solution. All membranes with DES addition showed higher PWF than the pristine PES membrane, achieving the maximum at 142.8 L m^−2^ h^−1^ with 2 wt% of the DES concentration.

**Figure 11:**
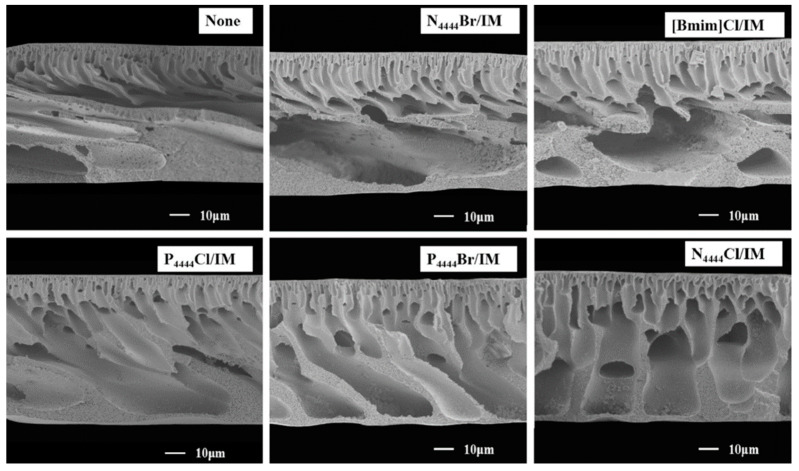
Cross-sectional SEM images of PES membranes with different IM-based DESs as additives. Reprinted from [128].

Also for UF, DESs based on the neutral organic molecule imidazole (IM) as the HBD combined and different organic salts as HBAs were synthesized by Jiang et al. [128] as novel pore formers. The first DESs studied was formed from tetrabutylphosphonium bromide ([P_4444_][Br]) as the HBA. The PES membranes were prepared at DES concentrations up to 4 wt% via NIPS, and the results showed a typical structure of a top-dense layer on a more porous sublayer. The addition of DESs contributed to an increase in the size of the macrovoids in the membrane when concentrations up to 2 wt% were used. The highest interconnection between pores was observed at a DES concentration of 2 wt%. Moreover, the ε increased from 48.2 to 83.6% at DES concentrations of 0 and 2 wt%, respectively, and the ε was then reduced at higher concentrations. Also, the mean pore size r_p_ showed similar behavior to that observed for the overall porosity ε, achieving a maximum r_p_ of 38.3 nm. The results obtained with [P_4444_][Br] and IM as DES components are related to the hydrophilicity and viscosity of the DES and solution. A high hydrophilicity of the additive would have accelerated the exchange ratio between the solvent and non-solvent, forming a more porous membrane. However, the increase in viscosity at high DES concentrations eventually reduced the DESs’ hydrophilicity action. DESs were also synthesized from tetrabutylammonium bromide ([N_4444_][Br]), 1-butyl-3-methyilimidazolium chloride ([BMIM][Cl]), tetrabutylphosphonium chloride ([P_4444_][Cl]), and tetrabutylammonium chloride ([N_4444_][Cl]) as HBAs. All of them were tested at the same concentration of 2 wt% for membrane preparation, in which the highest ε and r_p_ of 86.5% and 40.5 nm, respectively, were obtained with the [N_4444_][Cl]-based DESs. SEM images of all these membranes are given in Figure 11. The maximum pure water flux (PWF) was obtained by the membrane with a 2 wt% concentration of the DESs formed by IM and [P_4444_][Br], with a value of 651 L m^−2^ h^−1^ at the transmembrane pressure of 2 bar.

Also for UF, Kumar et al. [129] fabricated PES membranes by the NIPS method, using choline chloride (ChCl) and urea as components of the DES. The different molar ratios used were 1:2, 1:3, 1:4, and 1:5 for ChCl and urea, respectively, employing a 1 wt% DES concentration for all of them. No micropores on the surface could be seen for the membrane without DESs. However, the addition of DESs generated the appearance of pores on the surface and changes in the ε and the r_p_. The ε increased from 75 to 93%, and the r_p_ increased from 7.96 to 14.48 nm for membranes without DESs and 1 wt% DES concentration at a 1:3 molar ratio, respectively. With increasing the urea content in DESs, there was an enormous increase in water flux due to membrane morphology and hydrophilicity. The maximum water flux was seen for the 1:3 molar ratio membrane, with 233.9 L m^−2^ h^−1^.

**Figure 12:**
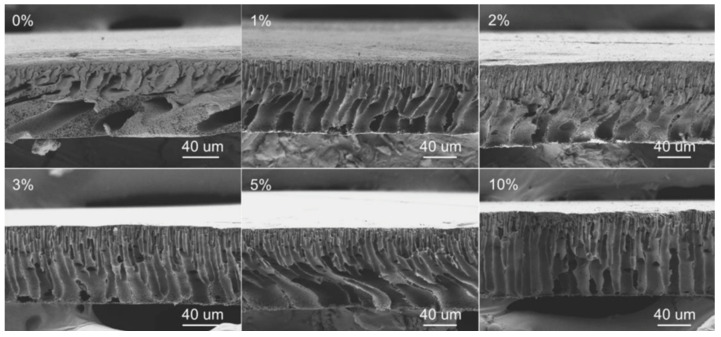
Cross-section SEM images for PSE membranes with different DES contents. Reprinted from [131].

To improve the permeability and selectivity in UF membranes, Zhou et al. [131] used ethylene glycol (EG) and ZnCl_2_ to form a DES for PSE membranes. The membranes were fabricated via NIPS, working from 0 to 10 wt% DES concentration. As the DES content increased from 0 to 3 wt% the average r_p_ decreased from 54.3 to 24.4 nm, and then increased to 40.9 nm when the concentration achieved 10 wt%, as shown in Figure 12. The pore density increased, and the r_p_ distribution became more uniform with a 3 wt% DES concentration. This indicated that blending DESs could tailor the surface pore structure on the membrane. In addition, when more DESs were added to the casting solution, some DES aggregation might form, resulting in a larger r_p_. In the case of morphology, all membranes showed a typical double-layer structure with a top dense layer and a macrovoid sublayer. However, the membranes without DESs showed a sponge-like bottom layer. The addition of DESs increased the polymer-lean phase content, and the prepared membranes became more porous, increasing the ε of the membrane from 73.2 to 84.0%, for 0 and 10 wt% DES concentration, respectively. This indicated the excellent pore-forming ability of the DES additive. Regarding the membrane’s performance, the PWF increased from 93.4 to 382.2 L m^−2^ h^−1^ for 0 and 10 wt% DES concentration, respectively. The enhancement of the membrane ε with the DES addition could be the reason for the PWF improvement.

**Figure 13:**
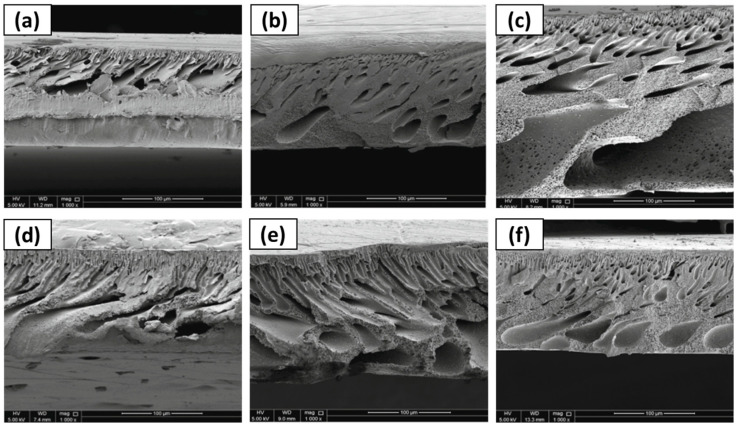
Cross-sectional SEM images of various synthesized FR-based DESs membranes. (**a**) PSF, (**b**) PSF/DES 1 wt%, (**c**) PSF/DES 1 wt%/PVP 3 wt%, (**d**) PSF/DES 2 wt%, (**e**) PSF/DES 3 wt%, and (**f**) PSF/DES 4 wt%. Adapted from [132].

PSF membranes were prepared by Elhamarnah et al. [132] for UF. The DES used as a pore former was formed by ChCl as the hydrogen bond acceptor (HBA) and fructose as the hydrogen bond donor (HBD) at a 1:1 molar ratio, studying concentrations between up to 4 wt%. The method employed for membrane formation was NIPS. Using this DES as a pore former resulted in significant changes in the membrane roughness. Also, the mechanical properties were affected, indicating that the addition of the DES to the membrane matrix enhanced the tensile strength of synthesized membranes. As shown in Figure 13, two distinct layers were encountered for all membranes, a dense top layer at the surface and a sponge-like structure at the bottom. This sub-layer comprised numerous finger-like macrovoids and tiny pores encased by the polymer wall. The addition of DESs promoted the development of a porous membrane with finger-like macrovoids that expanded and elongated its size due to the hydrophilic nature of DESs. Incorporating DESs into the membrane increased the pure water permeability across all concentrations. The ε and r_p_ showed increases in both parameters with rising DES concentration. The highest ε was exhibited by a membrane with 1 wt% DES concentration and poly (vinyl pyrrolidone) (PVP) presence, with 87.8% and a pore size of 4.405 nm. The maximum PWF obtained was 156 L m^−2^ h^−1^ for the membrane with 1 wt% of DES concentration and PVP content.

### 3.4. Other Uses of Deep Eutectic Solvents in Membrane Preparation

DESs have also been evaluated as a component of the non-solvent bath to modify the precipitation kinetics of the polymer for application in the separation of rare earth ions by static absorption. The work of Chen et al. [100] reported the use of DESs based on betaine and lactic acid (LA) in the coagulation bath for the preparation of PVDF-based membranes via NIPS. The focus of this study was based on the polarity of the DESs formed and its possible impact on the structure of the prepared membrane. The concentrations of DES in the coagulation bath ranged from 0 to 20 wt%. The surface of the studied membranes was analyzed by SEM, and the images revealed remarkable variations in their morphology with the modification of the concentration. The main appreciated differences were the significant increase in the pore and the diameter range for low levels of DES content, 1 and 5 wt%. However, after DES addition up to 10 wt% the pores became uniform and regular with an average size lower than 1.0 µm. In the cross-section of the prepared membranes, shorter and more vertical channels were observed when the DES concentrations were increased. This was attributed to an acceleration of the solidification process in the bath due to the increase in polarity generated by the DESs. This resulted in macrovoids inside the membrane at concentrations higher than 10 wt%. The PWF decreased with higher DES contents, which may for the migration of P=O and P-O groups to the lower epidermal layer of the membrane, making the membrane surface more hydrophilic but becoming more hydrophobic in the internal structure.

Finally, Liang et al. [101] prepared a new solvent-resistant triple-layer thin film composite (TFC) membrane on the polyimide substrate for forward osmosis applications. For this, the NIPS and IP methods were employed, and the DESs formed by cyclodextrin and malic acid at 1:5 and 1:10 molar ratios were used as an interlayer coating. The addition of DESs to the TFC membrane surfaces changed to be smoother and denser. The more viscous DESs, with a 1:10 molar ratio, made the interlayer uniformity poor, thus the roughness of the surface layer increased. In addition, the overall porosity (ε) did not suffer significant changes for DES coating. TFC membrane with DES coating at a 1:5 molar ratio showed an increase in the solvent flux to 9.51 L m^−2^ h^−1^.

## 4. Use of Deep Eutectic Solvents in Liquid Membranes

Liquid membranes were conceived as an enhancement of bare polymer membranes in terms of the mass transfer mechanism, which consists of a solution–diffusion process [141]. This makes liquid membranes a more versatile technology for different environmental applications, featuring separations in liquid or gas phase. 

The introduction of a liquid phase into the separation medium improves the tunability of the membrane, due to the wide range of liquid phases available to perform this functionality. In this regard, DESs offer a wide versatility and, thus, optimized task-specificity to perform a great variety of operations featuring environmental membrane technologies [29,41,102], especially emphasizing gas separation in applications such as biogas upgrading or CO_2_ elimination from industrial flue gases [29,41,102]. 

**Figure 14:**
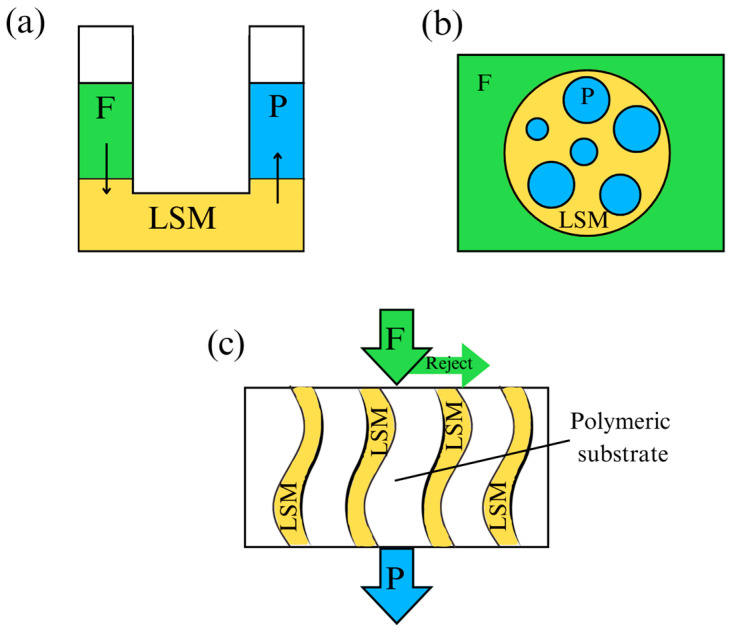
Scheme of the different types of liquid membranes: (**a**) bulk liquid membrane (BLM), (**b**) emulsion liquid membrane (ELM), and (**c**) supported liquid membrane (SLM). For each membrane, F refers to the feed introduced to the membrane, LSM is the liquid separating media which form each liquid membrane, and P is the permeate element(s) from the feed.

Liquid membranes can be found under three main different assemblies: bulk liquid membranes (BLMs), emulsion liquid membranes (ELMs), and supported liquid membranes (SLMs) [30]. All three structures are depicted in Figure 14. Additionally, Table 6 offers a general overview of the advantages and disadvantages of each type of liquid membrane.

Among all the possible liquid membranes, supported liquid membranes, SLMs, are the most popular ones due to their high mass transfer rate, permeability, and efficiency, since the liquid phase remains only in the pores of the polymeric phase [144,145,146] and is immobilized inside by means of capillary forces, as shown in Figure 14. The separation process performed by this membrane configuration can be understood as simultaneous extraction and stripping stages of the feed. In other words, one of the components, whichever has the stronger interactions with the liquid phase, is separated from the feed stream and, at the same time, a release of that component happens on the permeate side of the membrane [147]. This chapter is dedicated to the application of DESs in liquid membranes, especially as SLM carriers, in environmental applications.

**Figure 15:**
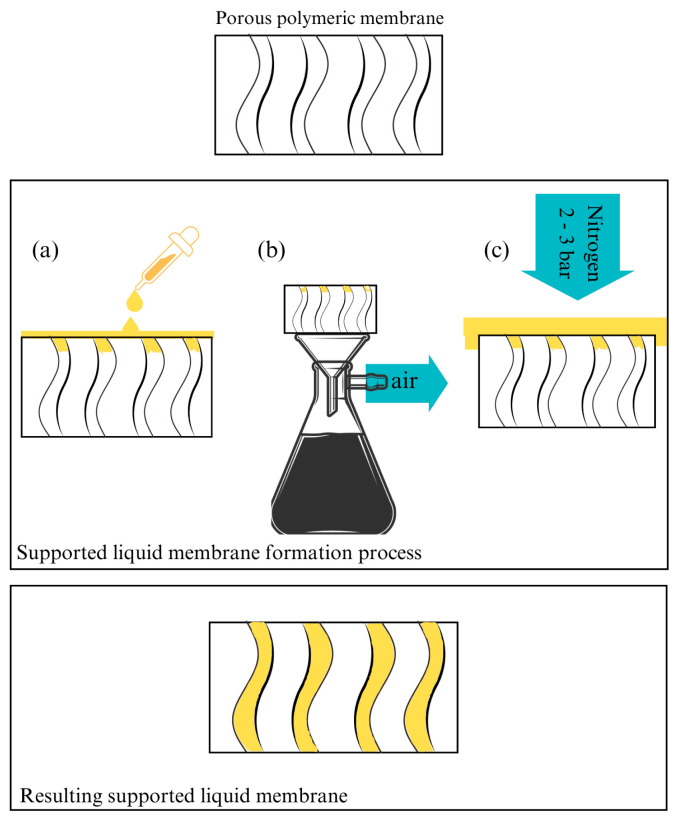
Supported liquid membranes preparation methodologies: (**a**) direct immersion, (**b**) vacuum method and (**c**) nitrogen pressure.

There are three common preparation methods of SLMs: direct immersion, pressure-driven, and vacuum-driven formation [148]. Direct immersion consists of soaking the membrane with the desired liquid additive at atmospheric pressure until the liquid is immobilized after a certain period. In contrast, the pressure-driven and vacuum-driven methods use a lower amount of liquid phase and vary the pressure of the process. In the case of the pressure-driven preparation, a gas such as N_2_ is incorporated into the impregnation chamber as an inert gas to force the DESs to enter the pores of the membrane at 2–3 bar; meanwhile in the vacuum-driven method, air is evacuated from the sample, facilitating the flow of the liquid additive across the membrane [149]. These methodologies are depicted in Figure 15, and the advantages and disadvantages of these preparation methods are gathered in Table 7.

Concerning stability, i.e., the resistance of the immobilized liquid in the pores to be swept away from the membrane during certain operations, such as gas–gas or gas–liquid separations, after the direct immersion method, the liquid is more prone to be dragged off, since the liquid phase enters only the most accessible pores inside the membrane. On the contrary, the pressure-driven and vacuum-driven methods offer more stability since the liquid additive is capable of penetrating the membrane pores deeper, being strongly immobilized within the polymer matrix.

**Figure 16:**
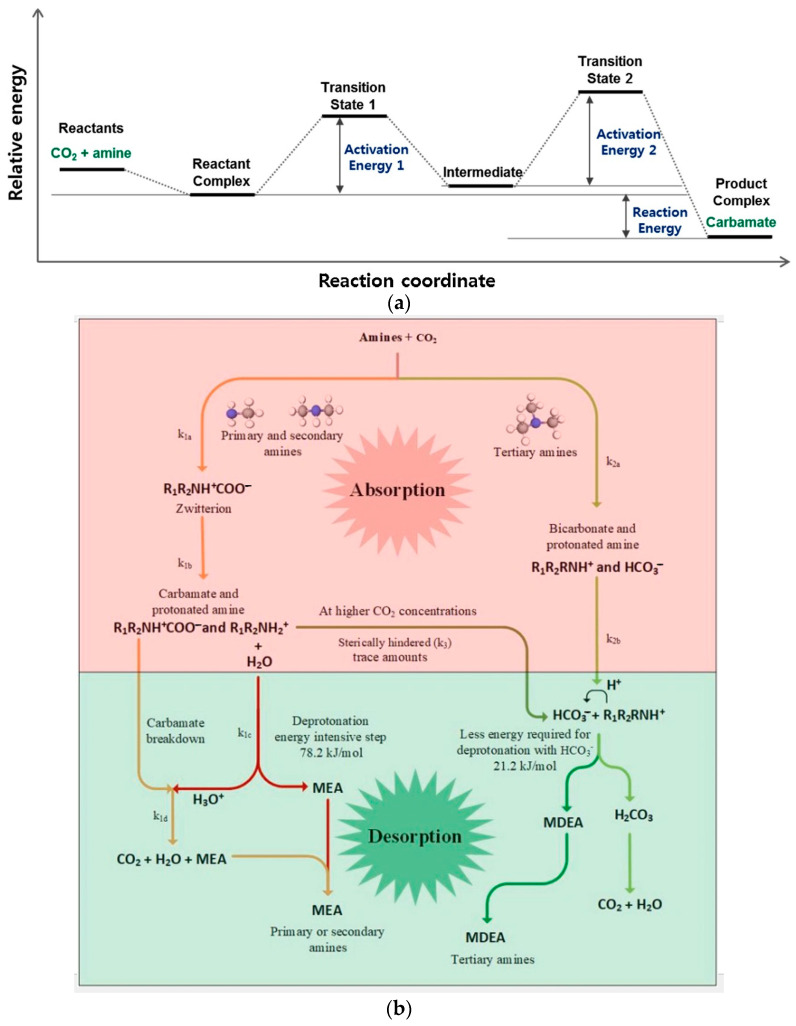
(**a**) Energy barriers for obtaining the carbamate complex as a result of CO_2_ and amine reaction. Reprinted from [151]. (**b**) Description of the desorption mechanism of CO_2_ from amines. Reprinted from [152].

DESs in SLM are believed to improve membrane’s separation by two main mechanisms: coupled counter-transport and coupled co-transport [28]. On the one hand, if the immobilized DESs has an acidic nature, such as those based on organic acids, e.g., decanoic, oxalic, malic acids, etc., the permeated molecule takes advantage of a coupled counter-transport. The acidic species release a proton to the system, which allows the charged component to complexate with the conjugate base. This phenomenon especially happens when acidic type III and IV DESs are used. Also, this mechanism allows the recovery of charged species such as metal ions [147]. On the other hand, coupled co-transport takes place when the DES species possess a basic or neutral nature. In this case, the complexation involves every species, and the transporting mechanism does not favor any component in particular. The charge of the species is not a significant driving force to increase selectivity towards a single species [147]. There must be other physicochemical interactions between feed species and DES components to increase the selectivity of the separation when using a neutral or basic DES HBD. These mechanisms appear because of the predominance of weaker interactions such as van der Waals interactions between DESs’ HBA, HBD, and carbon sources such as CO_2_. Contrarily, in amine absorption, the bonds between CO_2_ and, for instance, monoethanolamine or diethanolamine (MEA or DEA, respectively) are noticeably stronger than those observed in SLMs, requiring this way a significantly higher energetic input to carry out the process [23]. The breakage and formation of chemical bonds between these types of amines and CO_2_ provokes the appearance of intermediate species and increases the total activation energy of the amine regeneration process. As described in Figure 16a, to obtain again the initial reactants, the complexated amine and CO_2_ need to undergo two energetic barriers: one to achieve the formation of an intermediate zwitterion and a second one which results in a carbamate complex. The latter must be decomposed to obtain the original CO_2_, the amine, and a water molecule. This process is also described in Figure 16b.

**Figure 17:**
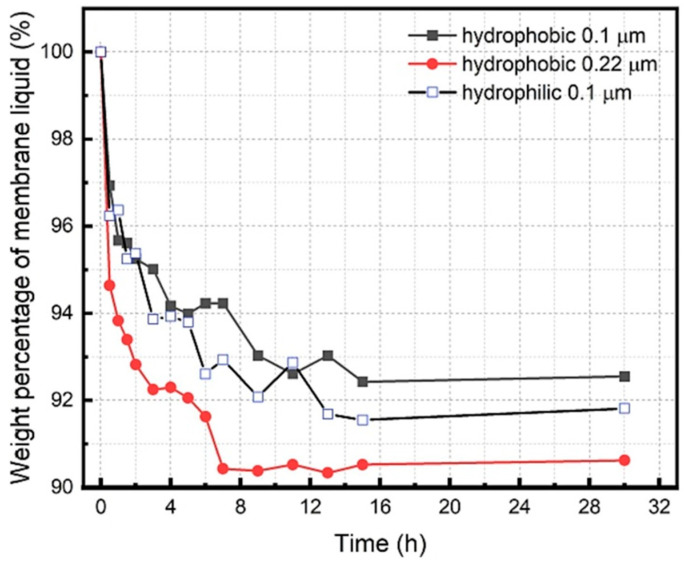
Relative liquid additive losses in three SLMs based on decanoic acid-lidocaine, as a function of time. Supports are different types of PVDF. Reprinted from [32].

Permeability, selectivity, and the aforementioned stability concepts also apply to the DES-based SLMs. Long-term stability is still a point in the works for some types of DESs, such as the newly introduced type V, since losses of additive have been reported in several works using decanoic acid and lidocaine DESs [134]. Additive losses, as described in Figure 17, almost reach 10% of the total additive inside a PVDF substrate at 7 h of CO_2_ and N_2_ gas separation experiment.

**Figure 18:**
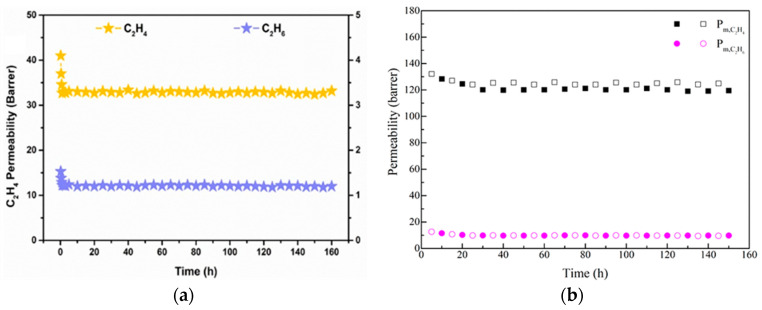
Time evolution of C_2_H_4_ permeability using PVDF SLMs based on (**a**) CuCl+ ethylamine hydrochloride:Glycerol DES (reprinted from [156]) and (**b**) CuCl+ChCl:ethylene glycol DES (reprinted from [157]).

As described before, the type III DESs generally includes molten salt species, which feature a cation and an anion that are liable to be complexated inside the DESs phase. This is the case of compounds such as ChCl, used as an HBA, mixed with organic HBDs, which usually feature organic acids. However, their long-term stability has been reported to be upgradable. To this respect, the addition of metallic compounds to type III DESs have achieved good results in terms of DES-based SLM’s long-term stability for gas separations [153,154,155], especially when those DESs are combined with species such as CuCl, as seen in Figure 18. Metallic salts demonstrate to maintain permeability values along extended periods, since both cases present an abrupt permeability change at the start of the operation, but this parameter remains constant until the end of both experiments.

Thus, the non-metallic interactions of some type III DESs presumably play an important role in the SLM stability, affecting the durability of the SLM [158]. Additionally, hydrophobic DESs are very interesting for SLM formation, since most of the membranes than can be obtained commercially tend to be hydrophilic. Thus, hydrophobic DESs may be successfully used in both gas separations and separations featuring a liquid phase, in addition to still displaying hydrogen bond interactions. Common hydrophobic DESs are based on menthol, thymol, decanoic acid, and other species consisting of aromatic groups and alkylic chains, which mainly conform to type V DESs, since these compounds exhibit non-ionic behavior [42,159].

In the field of decarbonization using DES-based SLMs, gas separations are the most extensive application found in the literature. Although DESs are being increasingly used in specialized research, other membrane technologies can be found in the decarbonization issue, such as mixed-matrix membranes (MMMs). MMMs are primarily based on the combination of matrixes of different natures, usually mixtures between polymeric and zeolitic species, with the possibility of also introducing liquid additives to these membranes. This typology of membranes has been chosen as benchmarking for SLMs and will not be further discussed in this review. Table 8 shows SLMs’ and MMMs’ permeability values and selectivity of CO_2_, N_2,_ and CH_4_, as well as the DESs used for their elaboration, specifying their composition in terms of the molar ratio.

**Figure 19:**
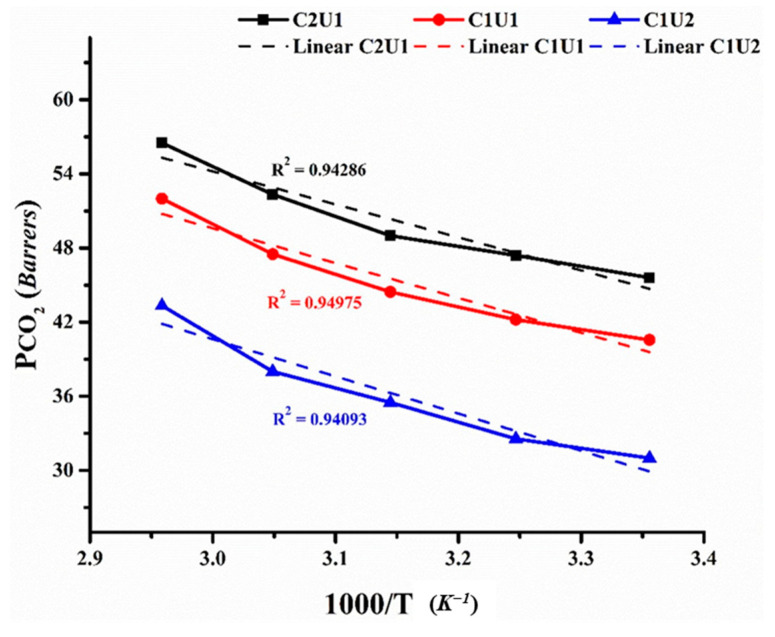
Temperature dependence of CO_2_ permeability of SLMs based on choline chloride-urea deep eutectic solvent at 2:1, 1:1, 1:2. Reprinted from [162].

Observing the DESs in Table 8, most of CO_2_ permeabilities look moderately low, specially when fluorinated membranes are used as the support material, which is the case of the PVDF and PTFE polymeric matrixes. On the contrary, organic materials that are cellulose- or PP-based tend to have a higher CO_2_ permeability. This can be connected to the viscosity of DESs, since they generally possess higher values than other liquid additives, namely conventional solvents and ionic liquids [178]. This phenomenon occurs especially for ChCl-based DESs, which explains why lower viscosity DESs such as Thy-Cou from [32] make an exception even using PVDF as a polymeric matrix support. Figure 19 shows the inverse proportionality between viscosity and permeability since an increase in temperature reduces the viscosity value and raises SLMs’ permeability.

The permeability values of SLMs shown in Table 8 are notably lower when observing CH_4_ or N_2_ performances, which makes these SLMs a very interesting decarbonization technology, as they demonstrated to be a highly selective media for CO_2_ separation. This affinity is produced by quadrupole–quadrupole interactions that occur when the oxygen atoms from the CO_2_ are attracted by the positive charges from either the DES species or the polymeric matrix carbon atoms, which also possess a positive charge, especially if the membrane features oxygen atoms on its backbone [179,180].

The positive charges produced in the DES or the membrane itself can be originated by several causes, depending on the DES and polymer type. For SLMs using ChCl as the HBA, the formation of a positive charge unit is due to the delocalization of the negatively charged halide species (Cl^−^) among HBD component of the DES. This circumstance is very common in systems such as ChCl-Urea 1:2 [181]. Also, for the newly introduced type V DESs, although these species are considered as non-ionic, the resonance effects exist, which increase the charges of the hydroxyl groups of species like thymol, making this group more positive than usual and creating a small polarization of the molecule [159]. This event also facilitates the transport of CO_2_ through the SLM, increasing its permeability. Lastly, MMMs have also been introduced in this study due to their novelty and the possible addition of neoteric liquid phases within a separation medium. This technology takes advantage of DESs [171], ionic liquids [174], and even amines [173]. The latter technology has been thoroughly investigated during the last century. Accordingly, complex processes featuring compounds possessing amino groups have been widely reported [173,182,183].

**Figure 20:**
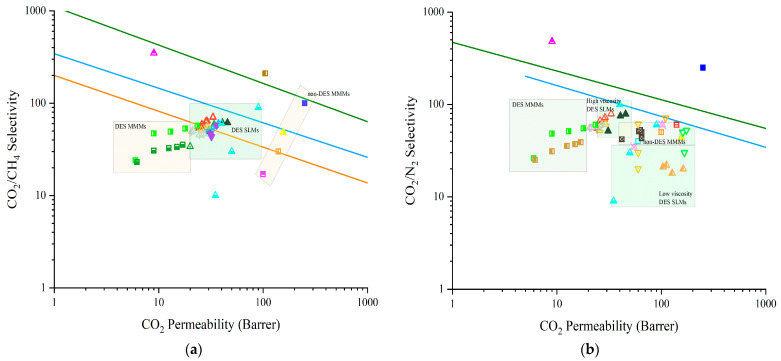
Upper bound plot comparing the permeability of CO_2_ with the selectivity of this gas for (**a**) CH_4_ and (**b**) N_2_. The represented lines are the upper bounds proposed by Robeson in 1991 (orange) and 2008 (blue) and by McKeown in 2019 (green). Also, these graphs differentiate between SLMs (circles) and MMM (squares). [161], hollow triangles; [162], solid triangles; [163], dot center triangles; [164], + center triangles; [34], - center triangles; [166], | center triangles; [167], half up solid triangles; [170], half right solid triangles; [184], x center triangles; [169], half left solid triangles; [165], inverse solid triangles; [32], inverse hollow triangles; [160], inverse dot center triangles; [27], half down solid triangles; [173], solid squares; [176], hollow squares; [174], - center squares; [175], | center squares; [172], half right solid squares; [171], half left solid squares; [177], x center squares.

To end this section, Figure 20 offers upper bound plots for the SLMs studied in Table 8, which show a representation of the well-known trade-off between the permeability and selectivity of SLMs and MMMs for the separation of CO_2_/CH_4_ and CO_2_/N_2_ gas pairs. In SLMs for CO_2_/N_2_ separations, there is a clear differentiation between achieving a high selectivity at the cost of a low permeability or vice versa, while most CO_2_/CH_4_ separation works register similar permeabilities and selectivity. Regarding DES-based MMMs, this newer technology still has room for improvement in these two parameters, despite having a similar performance to that of DES-based SLMs.

## 5. Concluding Remarks and Challenges of Deep Eutectic Solvents in Membrane Design, Functionalization and Applications

Deep eutectic solvents (DESs) and their implementation in polymer membranes for environmental applications emerged thanks to the outstanding tunability of properties, considering a wide combination of species at different relative ratios. DESs have been classified into five types according to the hydrogen bond acceptor (HBA) and hydrogen bond donor (HBD) involved in the DESs formation. In addition, the physicochemical properties of a great variety of DESs have been provided, namely melting temperature, surface tension, viscosity, and density.

Additionally, the most widespread methods in the fabrication of membranes have been described, highlighting the non-solvent induced phase separation (NIPS) method. Also, multiple works have been presented, where DESs were used as additives, solvents, co-solvents, or pore formers, among other uses, and the results were of great interest for future applications in membrane technology. Regarding the use of DESs in the manufacture of membranes by the NIPS method, the question arises as to the possible recycling and reuse of these compounds. Although the characteristics, concentration, and toxicity of DESs, along several economic factors, influence the most appropriate separation method, conventional evaporation is the most viable option for the separation of the non-solvent, usually water, when DESs is used as a solvent, [185,186,187]. However, if DES is used as an additive and, therefore, there is a different solvent in the process residue, the separation process requires new steps that are still under investigation. Membrane technologies such as dialysis, electrodialysis, or combined ultrafiltration–diafiltration–nanofiltration stages have been investigated, yielding promising results [188,189]. Most of the literature presented in this review includes sections where the modifications suffered by the polymer membrane were studied, focusing the presentation of results on variations in the porosity and internal morphology of the membranes. On multiple occasions, the addition of DESs to the manufacturing process significantly affected the overall porosity (ε) and pore size (r_p_), in addition to increasing the presence of finger-like structures and macrovoids in the cross-section. Performance data were also collected for membranes fabricated for different applications, with ultrafiltration (UF) and nanofiltration (NF) being the most relevant. In addition, a special focus on supported liquid membranes (SLMs) is given. The DES-based SLMs preparation methods are also discussed in this work. Finally, the permeability and selectivity values of different gases, such as CO_2_, N_2,_ and CH_4_, have been collected for liquid membranes supported on substrates of diverse natures.

Membrane technologies have demonstrated that they have a brilliant future regarding their use in environmental applications. Also, deep eutectic solvents (DESs) have enhanced the production and functionalization processes of membranes. However, they still have issues that need to be addressed to unlock their full potential. The following lines explore promising alternatives for the use of DESs in the field of polymer membranes:

- Regarding the use of DESs during membrane preparation, DESs could also be used as a substitute of the non-solvent component of the non-solvent induced phase separation (NIPS) process since the polarity of some DESs has been widely reported [98].
- Enhancing the retention of DESs inside supported liquid membranes (SLMs) is fundamental for the success of any operation. The most important step is to improve the low stability of the developed SLMs. The high DESs loss that most of the studied DES-based SLMs suffer needs to be palliated by moving onto new types of DESs. Ideally, attraction between DESs and CO_2_ should be stronger than van der Waals bonds, but still reversible as non-covalent bonds.
- Metal-based DESs (types I, II, and IV) offer a tailored design for the gas separation application without losing the characteristic advantages of supported liquid membranes (SLM) technology [190]. This tunability englobes forementioned physicochemical properties of the DESs such as viscosity or surface tension and also chemical affinity of the additive towards the gas to separate.
- On this line, innovative DESs might also be developed to be used for both pore-forming and SLM applications. DESs based on natural resources like sugar or amino acids [191] might be adequate for these purposes as renewable and solvents. Alternatively, membrane materials can be designed for DESs to modify them. Such is the case of graphene oxide (GO) matrixes, whose interlayer space can be tuned depending on the amount of DESs applied for the SLM fabrication, potentially varying its permeability [167].
- The use of DESs in the synthesis of membranes, either as pore formers or co-solvents, should explore new fields of environmental applications: The pore-forming ability of these additives may be controlled until tailoring nanopores for separations in gas phase or separations of gas from liquid effluents. The latter application could also be explored with DES-based SLMs. The removal of methane [192] or phosphorus [17] from liquid effluents is a field that needs to be further developed with the help of DES-based SLMs. Non-ionic (type V) DESs could be an answer to this aspect and a new niche to discover with this technology.
- Also, in this respect, further functionalization of membranes, before or after the formation of DES-based SLMs, may constitute a pathway of future investigation in this field. Apart from performing DESs immobilization within the membrane’s pores, additional functionalization might be implemented by grafting silicon-based compounds on the surface of the membrane, improving its hydrophobicity and opening new processes to perform decarbonization operations [193].
- Finally, on an environmental perspective, life cycle analysis of the developed DES-based SLMs should be performed to assess the impact of these products to the ecosystem in terms of biodegradability of the employed membranes and toxicity of the DESs that has been introduced, especially on a post-service point of view.

## Figures and Tables

**Table 1:** Compounds commonly used as hydrogen bond acceptor (HBA) and hydrogen bond donor (HBD) for deep eutectic solvent (DES) formulation.

HBA	Chemical Structure	HBD	Chemical Structure
Choline Chloride (ChCl)	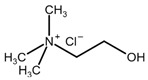	Urea	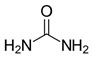
Betaine	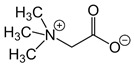	Glycerol	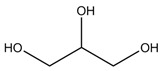
ZnCl_2_	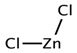	Ethylene Glycol	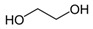
Thymol	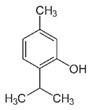	Decanoic acid	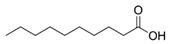
Tetrabutylammonium Bromide ([N_4444_][Br])	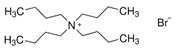	Oxalic Acid	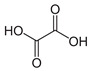

**Table 2:** Deep eutectic solvents classification by types regarding the nature of HBAs and HBDs.

Type	HBA	HBD
I	Ammonium, phosphonium, metallic salts, and other compounds that can be classified as [C^m+^]_n_ [A^n−^]_m_	Metallic halides
II	Ammonium, phosphonium, metallic salts, and other compounds that can be classified as [C^m+^]_n_ [A^n−^]_m_	Hydrated metal halides
III	Ammonium, phosphonium, metallic salts, and other compounds that can be classified as [C^m+^]_n_ [A^n−^]_m_	Compounds containing amides (CONH_2_), carboxylic (COOH), or hydroxyl (OH) groups.
IV	Metallic halides	Compounds containing amides (CONH_2_), carboxylic (COOH), or hydroxyl (OH) groups.
V	Non-ionic compounds	

**Table 3:** Main physicochemical properties of deep eutectic solvents commonly used in the literature, classified by type.

Type	HBA	HBD	Molar Ratio	Melting Point (K)	Surface Tension (mN·m^−1^) (298 K)	Viscosity (mPa·s) (298 K)	Density (g·cm^−3^) (298 K)	Refs.
I	ChCl	ZnCl_2_	1:2	325.3		415		[44]
I	ChCl	ZnCl_2_	1:3	320.4		57.5		[44]
I	[P_4444_][Br]	ZnCl_2_	1:2	341.0		2359		[44]
I	[P_4444_][Br]	ZnCl_2_	1:3	343.0		6196		[44]
II	ChCl	[CrCl_3_·6H_2_O]	2:1			9074	1.38	[45]
II	ChCl	[CrCl_3_·6H_2_O]	1:2			4819	1.58	[45]
III	[P_4444_][Cl]	Levulinic Acid	1:2			167	1.02	[46]
III	[MTP][Br]	Ethylene Glycol	1:1		47.5			[47]
III	[N_1111_][Cl]	Lactic Acid	1:2			756	1.14	[48]
III	[N_2222_][Cl]	Lactic Acid	1:2			441	1.11	[48]
III	[N_3333_][Br]	Glycerol	1:2		51.6			[49]
III	[N_3333_][Br]	Ethylene Glycol	1:3		46.6			[49]
III	[N_4444_][Cl]	Lactic Acid	1:2			890	1.02	[48]
III	[N_4444_][Br]	Acetic Acid	1:1		34.5			[47]
III	[N_4444_][Br]	Oxalic Acid	1:1		42.7			[47]
III	Betaine	Urea	1:2	359				[50]
III	Betaine	Ethylene Glycol	1:3		56	36	1.13	[51,52,53]
III	Betaine	Glycerol	1:2				1.22	[53]
III	ChCl	Urea	1:2	285	52	828	1.19	[54,55,56,57]
III	ChCl	Ethylene Glycol	1:2	237				[58]
III	ChCl	Ethylene Glycol	1:3		59	31	1.11	[51,53,59]
III	ChCl	Glycerol	1:2	233	57.2	400	1.19	[53,60,61]
III	ChCl	Glucose	1:2			8000		[62]
III	ChCl	Glucose	1:1		73	9000	1.27	[62,63]
III	ChCl	Glucose	2:1		71.6			[62]
III	ChCl	D-Fructose	1:1	293				[64]
III	ChCl	D-Fructose	2:1	283				[64]
III	ChCl	Citric Acid	1:2	344				[65]
III	ChCl	Lactic Acid	1:1			1160	1.17	[66]
III	ChCl	Lactic Acid	1:1 +20% water			25	1.15	[66]
III	ChCl	Lactic Acid	1:2		47.4			[67]
III	ChCl	Lactic Acid	1:4		44.4			[67]
III	ChCl	Levulinic Acid	1:2		39.4			[47]
III	ChCl	Malic Acid	1:1.5	325				[65]
III	ChCl	Malic Acid	1:1			11,000	1.27	[66]
III	ChCl	Malic Acid	1:1 +20% water			60	1.23	[66]
III	ChCl	Malonic Acid	1:1	283	65.7	1389	1.23	[68,69]
III	ChCl	Oxalic Acid	1:1	307		8953	1.26	[66,68,69]
III	ChCl	Tartaric Acid	1:2	310				[70]
III	ChCl	Tartaric Acid	2:1			89,967	1.18	[70]
III	ChCl	O-cresol	1:3				1.07	[71]
III	ChCl	Phenol	1:3				1.09	[71]
III	Menthol	Octanoic Acid	1:1		26.7			[47,72]
III	Menthol	Decanoic Acid	1:1			16	0.89	[72]
III	Thymol	Octanoic Acid	1:1		29.1			[47]
III	Thymol	Decanoic Acid	1:1	285		12	0.93	[72,73]
III	Thymol	Decanoic Acid	1:2		28.4			[47]
IV	CoCl_2_· 6 H_2_O	Lactic Acid	1:6	235.9	35	160	1.34	[74]
IV	NiCl· 6 H_2_O	Urea	1:2	235.3	89.9	500	1.58	[74]
IV	ZnCl_2_	Lactic Acid	1:1				1.12	[75]
V	Thymol	Menthol	2:1	285		27	0.95	[72,76]
V	Thymol	Menthol	1:1	257		37	0.93	[72,76]
V	Thymol	Menthol	1:2	267		44	0.92	[72,76]
V	Thymol	Urea	1:2	268				[77]

**Table 6:** Comparison of the different liquid membrane configurations.

Type of Configuration	Overview	Advantages	Disadvantages	Ref.
Bulk Liquid Membranes (BLM)	U-shaped tube with 3 phases: a feed and a receiving phase in contact with a membrane phase	Good for preliminary selectivity and kinetic transfer measurements High stability	Very low surface area Very low scalability	[142]
Emulsion Liquid Membranes (ELM)	Double emulsion: acceptor inside a membrane phase, which strips the feed	High recovery of metal species High mass transfer	High number of parameters to control Difficult stability control	[143]
Supported Liquid Membranes (SLM)	Polymeric membrane with pores filled with a liquid phase, which facilitates transport	Low cost Easy preparation High mechanical stability	Possible sweep of liquid phase Must operate at low pressures	[30]

**Table 7:** Overview of laboratory-scale supported liquid membrane preparation methods at laboratory scale.

Method	Advantages	Disadvantages	Refs.
Direct immersion	Easy to perform Low cost	Time-consuming Not valid for viscous DESs	[150]
N_2_ pressure	Ensures impregnation of all DESs Short immobilization time	Requires a more complex setup	[34,146,147]
Vacuum	High pore filling Simple assembly	Higher DESs consumption Requires vacuum pump	[32]

**Table 8:** Examples of different liquids used for supported liquid membranes elaboration, with their permeability and selectivity values.

Membrane Support	Carrier	Molar Ratio	$\mathrm{Permeability}\;{\mathrm{CO}}_{2}\;(P_{{CO}_{2}})$ (Barrer)	$\mathrm{Permeability}\;{\mathrm{CH}}_{4}\;(P_{{CH}_{4}})$ (Barrer)	$\mathrm{Permeability}\;N_{2}\;(P_{N_{2}})$ (Barrer)	$\mathrm{Selectivity}\;{\mathrm{CO}}_{2}\;(\alpha_{{CO}_{2}/{CH}_{4}})$	$\mathrm{Selectivity}\;{\mathrm{CO}}_{2}\;(\alpha_{{CO}_{2}/N_{2}})$	Ref.
Supported liquid membranes (SLMs)								
PTFE	ChCl:Urea	1:2	90.0	1.0	1.7	90.0	60.0	[27]
	ChCl:Gly	1:2	40.0	0.7	0.4	60.0	100.0	
	ChCl:EG	1:2	50.0	1.7	1.7	30.0	30.0	
	ChCl:OxA	1:2	35.0	3.5	3.9	10.0	9.0	
	ChCl:LA + 4% water	1:2	110.0		1.5		71	[160]
	ChCl:LA + 9% water	1:2	60.0		1.0		61	
	ChCl:LA + 27% water	1:2	60.0		2.0		30	
	ChCl:LA + 36% water	1:2	60.0		3.0		20	
PVDF	ChCl-MEA	1:8	32.2	0.45	0.41	70.5	78.4	[161]
	ChCl-DEA	1:8	27.7	0.44	0.40	63.0	69.3	
	ChCl-Urea	2:1	43.5	0.72	0.58	60.4	73.4	[162]
	K_2_CO_3_-Gly	1:6	34	0.58	-	59	-	[163]
	ChCl-OxA	1:1	35.5	0.61	-	58.6	-	[164]
	ChCl-Urea	1:1	38.0	0.65	0.52	58.5	73.9	[162]
	Bet-MalA	1:1	29.3	0.52	0.48	56.4	61.1	[34]
	Bet-Urea	1:3	33.4	0.6	-	55.7	-	[165]
	ChCl-T E A	1:8	25.1	0.46	0.42	54.6	59.8	[161]
	ChCl-MalA	1:1	33.3	0.61	-	54.5	-	[164]
	Be-TartA	1:1	25.6	0.5	0.49	51.1	52.1	[34]
	ChCl-PAA	20:1	21.3	0.42	0.38	50.7	56.1	[166]
	ChCl-PAA	15:1	20.6	0.42	0.37	49.0	55.6	
	ChCl-TartA	1:1	27.5	0.56	-	49.0	-	[164]
	ChCl-Urea	1:2	29.4	0.63	0.6	46.7	49.2	[162]
	ChCl-PAM	20:1	27.0	0.58	0.45	46.6	60.0	[166]
	ChCl-PAM	15:1	24.5	0.54	0.43	45.4	57.0	
	Bet-Gly	1:3	29.3	0.65	-	45.1	-	[165]
	Bet-EG	1:3	30.5	0.72	-	42.3	-	
	K_2_CO_3_-EG	1:6	20	0.59	-	34	-	[163]
	Thy:Cou + 20% NFM	1:1	161		3.2		49.9	[32]
	Thy:Cou + 40% NFM	1:1	174.1		3.3		52.4	
	Thy-Cou	1:1	166.6	-	5.55	-	30.0	
GO	ChCl-EG	1:4	9	0.02	0.02	~350	~480	[167]
Ti-Nanosheets	ChCl-EG	1:4	105.0	0.5	0.42	210	250	[168]
PP	ChCl-1,2-propanediol	1:3	104	-	3	-	29	[169]
		1:4	112	-	5	-	22	
Cellulose nanofibers	ChCl-ZnCl_2_	1:2	155.8	3.2	3.6	48.4	43.6	[170]
Polymer membranes								
PSF			6.2	0.3	0.2	23.0	25	[171]
PSF/THF			23.5	0.11	0.10	57	60	[172]
Mixed-matrix membranes (MMM)								
PVA + amine + HMMP-1 nanoparticles MMM			~250	~2.5	~1	~100	~250	[173]
Pebax^®^ + SSMMP% + [BMIM][NTf_2_] MMM			140	4.7	2.3	30	60	[174]
[N_4444_][l-prolinate] + pebax			100	6	2	17	50	[175]
Pebax^®^ + Zeolite imidazole framework MMM			60		1.5		40	[176]
[N_16,1,1,1_][Br][AcH] − Ceria 2% + PSF MMM			9.0	0.3	0.3	30.5	31	[171]
[N_16,1,1,1_][Br]:[AcH] − Ceria 4% + PSF MMM			12.5	0.4	0.4	32.5	35.5	
[N_16,1,1,1_][Br]:[AcH] − Ceria 6% + PSF MMM			15.0	0.4	0.4	33.5	37	
[N_16,1,1,1_][Br]:[AcH] − Ceria 8% + PSF MMM			17.0	0.5	0.4	35.5	39	
PSF/THF + ChCl:DecA 1:1/SBA 5%			18	0.17	0.16	53	55	[172]
PSF/THF + ChCl:DecA 1:1/SBA 10%			13	0.27	0.25	49	51	
PSF/THF + ChCl:DecA 1:1/SBA 15%			9	0.38	0.38	47	48	
L-arginine EG 1:5 Pebax^®^ 0%			65.0		1.5		43	[177]
L-arginine EG 1:5 Pebax^®^ 5%			65		1.3		50	
L-arginine:EG 1:5 Pebax^®^ 10%			61.0		1.2		51	
L-arginine:EG 1:5 Pebax^®^ 15%			63.0		1.2		52.5	
L-arginine:EG 1:5 Pebax^®^ 20%			42.0		1		42	

## List of Symbols and Abbreviations

(1S)+B3:B28-(+)-10-camphorsulphonic acid	(+)CSA
1-butyl-3-methyl-imidazolium bromide	[BMIM][Br]
1-butyl-3-methyl-imidazolium chloride	[BMIM][Cl]
1-ethyl-3-methyl-imidazolium chloride	[EMIM]Cl
Tetramethylammonium species	[N_1111_] ^+^
Tetraethylammonium molecules	[N_2222_] ^+^
Tetrabutylammonium molecules	[N_4444_] ^+^
Tetrabutylammonium bromide	[N_4444_][Br]
Tetrabutylammonium chloride	[N_4444_][Cl]
Tetrabutylphosphonium bromide	[P_4444_][Br]
Tetrabutylphosphonium chloride	[P_4444_][Br]
Acetamide	AA
Acetone	AC
Acetic acid	AcA
Betaine	BET
Bulk liquid membranes	BLMs
Citric acid	CA
Co-solvent-assited interfacial polymerization	CAIP
Casting evaporation	CE
Methane	CH_4_
Choline chloride	ChCl
Carbon dioxide	CO_2_
Coumarine	Cou
Chitosan	CS
Diethanolamine	DEA
Decanoic acid	DecA
Deep Eutectic Solvent	DES
Polymeric deep eutectic solvents	DESP
Nanoparticles ethaline coated	DES-SiO_2_
N,N-Dimethyl acetamide	DMAc
Dimethyl formamide	DMF
Dimethyl sulfoxide	DMSO
Ethylenediaminetetraacetic acid	EDTA
Ethylene glycol	EG
Emulsion liquid membranes	ELMs
Electrospinning	ES
Choline chloride: ethylene glycol (1:2) deep eutectic solvent	Ethaline
Ethanol	EtOH
Fructose	FR
Greenhouse gases	GHGs
Glucose	GLU
Glycerol	GLY
Glycolic acid	GLYA
Graphene oxide	GO
Gas separation	GS
Hydrogen bond acceptor	HBA
Hydrogen bond donor	HBD
Itaconic acid	IA
Ionic Liquids	ILs
Imidazole	IM
Interfacial polymerization	IP
Isopropanol	IPA
DL-lactic acid	LA
Levulinic acid	Lev
L-malic acid	MA
Malic acid	MalA
Monoethanolamine	MEA
Microfiltration	MF
Mixed-matric membranes	MMMs
Membrane preparation technique	MPT
Nytrogen	N_2_
Natural Deep Eutectic Solvents	NADESs
Nanofiltration	NF
4-formyl-morpholine	NFM
Non-solvent induced phase separation	NIPS
N-methyl acetamide	NMA
N-methyl-pyrrilidone	NMP
N-methyl urea	NMU
N,N-dimethyl urea	NNDMU
Non-solvent induced phase separation with temperature gradient	NTIPS
Oxalic acid	OxA
Polyamide	PA
Polyacrylic acid	PAA
Propionic acid	PAc
Polyamide-imide	PAI
Polyacrylamide	PAM
Polyacrylonitrile	PAN
Protonated 2-pyrrolidone-5-carboxylic acid	PCA
Permeability of methane	P_CH4_, Barrer
Permeability of carbon dioxide	P_CO2_, Barrer
Poly (ethylene glycol)	PEG
Polyethersulfone	PES
Phenyl acetic acid	PhAA
Permeability of nitrogen	P_N2_, Barrer
Polypropylene	PP
Protonated L-proline	PPRO
L-proline	PRO
Polysulfate	PSE
Polysulfone	PSF
Polytetrafluoroethylene	PTFE
p-toluensulphonic acid	PTSA
Pervaporation	PV
Polyvinylidene fluoride	PVDF
Poly (vinyl pyrrolidone)	PVP
Pure water flux	PWF, L m^−2^ h^−1^
Radius of the average size-void	r, m
Mean roughness	R_a_, nm
Reverse osmosis	RO
Pore size	r_p_, nm
Root mean square roughness	R_q_, nm
3-(N,N-dimethybutyammonio) propane-1-sulfonate	SB3-4
(3-(1-methyl-1H-imidazole-3-ium-3-yl) propane-1-sulfonate)	SB3-MIM
Scanning Electron Microscopy	SEM
Supported liquid membranes	SLMs
Tartaric acid	TarA
Benzyl-trimethylammonium mesylate	TBnA MsO
Triethyl phosphate	TEP
Thin film composite	TFC
Thymol	Th
Trimethyl glycine	TMG
Ultrafiltration	UF
Static water contact angle	WCA, º
Selectivity of carbon dioxide with respect methane	α _CO2/CH4_
Selectivity of carbon dioxide with respect nitrogen	α _CO2/N2_
β-cyclodextrin	β-CD
Surface tension	γ
Overall porosity	ε, %
